# Analysis of insecticide-treated bednet market dynamics between 2004–2021 and monetary value of additional bednet longevity

**DOI:** 10.1186/s12962-026-00725-0

**Published:** 2026-02-25

**Authors:** Amanda McCoy, Edward Thomsen, Angus Spiers, Eve Worrall

**Affiliations:** 1 Liverpool School of Tropical Medicine, Clinical Sciences Department, Global Health Economics and Financing Unit, Pembroke PL, Liverpool, L3 5QA UK; 2 Innovation to Impact, Liverpool School of Tropical Medicine, Pembroke PL, Liverpool, L3 5QA UK; 3 Malaria Elimination Initiative, Institute for Global Health Sciences, University of California, San Francisco, CA USA; 4 Oxitec Ltd, 71 Innovation Dr, Milton Park, Abingdon, OX14 4RQ UK

**Keywords:** Insecticide treated nets, Competition in the ITN market, ITN market inefficiency, Equivalent annual cost, Willingness-to-pay (WTP), Value-based procurement

## Abstract

**Background:**

Insecticide-treated bednets (ITNs), a cornerstone of malaria prevention, are distributed via mass campaigns across Africa every three years, at huge cost to National Ministries of Health and development partners. While WHO sets global standards for ITNs as a public health commodity, they typically remain in use for less than the assumed three years, due to accumulation of damage, and retention times vary according to the product and use context. However, it is currently unclear whether ITN prices reflect their value in terms of physical durability.

**Methods:**

We explored how various ITN product and market characteristics have influenced real ITN prices since 2004. We used ITN price and retention data across sub-Saharan Africa to calculate the country specific equivalent annual cost of ITNs and defined country-specific price thresholds that the market should be willing-to-pay to secure nets that would be retained and used for exactly three years or, separately for six months longer than current estimates suggest.

**Findings:**

The ITN market has become less concentrated in the last two decades, but it remains dominated by a few large buyers and suppliers. ITN prices have decreased dramatically since 2010. Among individual sales, we found no evidence that increased durability is rewarded through higher price. The value for equivalent annual cost per person protected and the willingness-to-pay for a net that is retained for longer depends on baseline net price and is greater in countries with shorter existing ITN retention times.

**Interpretation:**

Substantial public health and efficiency gains could be realized if bednets were used for longer periods. However, achieving this requires changes in the market on both the supply and demand sides, including a shift towards value-based procurement. This approach would better incentivize manufacturers to innovate and invest in producing more durable nets by linking pricing to their long-term effectiveness. An explicit price threshold, along with improved information on net durability across different contexts, could help foster innovation and direct investment where it is most needed.

**Supplementary information:**

The online version contains supplementary material available at 10.1186/s12962-026-00725-0.

## Key points for decision makers

Though ITN market competition increased and prices fell in the last two decades, inefficiencies remain in this market.

While ITN prices appear to reward chemical innovation, there is no evidence to suggest that they reflect the physical durability of the nets.

Information asymmetry related to the physical durability of ITNs has potential to be reduced by a newly developed key performance metric, however market and price incentives will also be needed to encourage manufacturers to improve the physical durability of ITNs

Value-based procurement provides an opportunity to set ITN prices that adequately reflect buyer’s willingness-to-pay for improved physical durability as well as chemical performance attributes, and to incentivise manufacturers to invest in improving physical durability

Durability should be regarded as a critical component in defining the “value” of insecticide-treated nets (ITNs), and a measure of resistance to damage has been developed to evaluate this aspect.

**Supplementary information:**

The online version contains supplementary material available at 10.1186/s12962-026-00725-0.

## Background

Malaria remains a major global health challenge, with cases reaching a record 263 million and annual deaths rising from 562,000 in 2015 to 597,000 in 2023 [1, 2]. Key obstacles to malaria control include insecticide and drug resistance, climate-related disruptions, and persistent funding gaps—only $4 billion was available in 2023, far short of the $8.3 billion required to meet control targets.

Insecticide-treated nets (ITNs) have been a cornerstone of global malaria control, particularly in sub-Saharan Africa, where they were responsible for averting 68% of all cases between 2000 and 2015 [3]. Consistently shown to be cost-effective [4], ITNs gained further traction in 2007 when the World Health Organization (WHO) endorsed them as a public health good [5], leading to major funding increases and widespread distribution—over 3 billion ITNs have been distributed globally since 2004, with 93% of the 254 million nets delivered in 2022 going to sub-Saharan Africa [1]. Despite this scale-up, progress, has stalled in recent years, underscoring the urgent need for continued innovation and investment in malaria control strategies. [1, 2]

Growing need and limited resources have resulted in greater unmet demand and an associated decrease in ITN access since 2017 [6]. At the same time, decreasing ITN effectiveness has become a key challenge [7]. ITN product development and innovation has focused primarily on chemical treatments to counter insecticide resistance [7]. Notable innovations on the original pyrethroid only ITN include ITNs that contain a synergist called piperonyl butoxide (PBO) to boost insecticide effectiveness of the insecticide (known as PBO nets), as well as nets with two active ingredients (dual AI nets). However, physical durability also impacts ITN effectiveness. Wear and tear leads to holes and other damage that significantly reduces ITN effectiveness; even WHO-prequalified nets have been found to have poor durability [8–10]. Poor physical durability also reduces the useful life of ITNs, with most being discarded before their target three-year lifespan due to damage [11]. This results in coverage gaps between the three yearly ITN coverage campaigns, with some countries considering increasing the frequency of ITN distribution campaigns (with substantial logistical costs) to ensure consistent protection. In the context of increasing scarcity, these inefficiencies can be ill afforded, yet limited investment has been directed toward improving physical integrity, durability and value of ITNs [12].

### Shaping ITN markets: competition, value, and innovation

A ‘perfectly competitive’ market is a hypothetical model where resources are allocated efficiently to maximise satisfaction to society [13]. No market is perfectly competitive, but comparing real-world market qualities with the conditions of perfect competition allows us to identify potential areas to shape current markets and improve efficiency. Though the impact of competition in healthcare has long been debated [14], information on its impact in the ITN market is lacking. According to economic theory, market conditions combine with technological know-how to generate innovation. Competition between numerous producers is required to incentivise innovation for the purpose of capturing additional market share and/or allowing them to charge a price-premium for a differentiated product, generating profit that can be channelled into further innovation. On the demand side, buyers must have information available that allows them to differentiate between high and lower value products and be willing to pay a premium price for a more valuable product. In the public health market for ITNs, normative bodies like the World Health Organisation (WHO) play a key role in providing information to buyers, through quality assuring products for example through pre-qualification processes. The ITN pre-qualification process is currently coordinated by the WHO PQ-VCT [15], and was previously under the remit of the WHO Pesticide Evaluation Scheme (WHOPES) [16].

A central challenge in the ITN market is the disconnect between those who purchase ITNs and those who ultimately use them. Global health partnerships and donor agencies often purchase and procure products on behalf of national governments, who then distribute them to end-users. This separation between purchasers and beneficiaries weakens the link between product performance and price. Although value-based procurement—where purchasing decisions reflect product quality, effectiveness, and longevity rather than unit cost alone—are gaining traction [17], its application in ITN procurement remains limited. Key challenges include political and logistical constraints, the absence of practical, country-specific indicator for ITN value, and limited capacity to operationalise such metrics in procurement decisions.

As a result, procurers lack clear guidance on how much more they should be willing to pay for higher-performing products, even where monopsonistic purchasing power could be used to reward value rather than volume. In this paper we seek to describe how competition and concentration among ITN sellers and buyers, along with other factors, may have impacted the market price of ITNs between 2004 and 2021, possibly contributing towards a lack of investment in development and production of more durable ITNs. In this context, market concentration refers to the extent to which a small number of firms account for a large share of total ITN sales, and is commonly measured using concentration ratios or the Herfindahl–Hirschman Index (HHI) [18].

We also explore an indicator of ITN value that accounts for the duration of protection, the number of people protected, and the price paid. Using country-specific data, we calculate this indicator and estimate the value of a price premium for more durable nets, highlighting that countries with higher malaria burden or shorter net lifespans stand to gain the most from such approach.

## Methods

### Market analysis

#### Data sources

ITN transaction data from 2004 to 2021 were obtained from the Global Fund [19] and the erstwhile President’s Malaria Initiative (PMI) [20]. Data were cleaned and merged to facilitate analysis. Given that ITNs are made from plastics such as polyester, polyethylene and polypropylene, the average price of oil was used as a proxy for ITN raw materials cost. All costs are in USD and have been adjusted for inflation using World Bank data (see Online Resource). Transaction data were compared to ITN distribution data that was publicly available at the time of our analysis, to assess completeness against estimated total market size [21]. All data manipulation is reported according to the Guidelines for Accurate and Transparent Health Estimates Reporting [22].

#### Data analysis

All analyses were completed in SPSS V 28.0. Basic descriptive statistics were computed and visualised through box and whisker plots, showing medians and inter-quartile range by net type. An extreme outlier was excluded from the analysis (a PBO net priced at 84USD in 2019), as were transactions considered to be of untreated nets.

##### Concentration/Competition

Two measures of ITN market concentration were computed: the Herfindahl-Hirschman Index (HHI) and the concentration ratio.

The HHI ranges between 0 (perfect competition with no supplier having a very large market share) and 10,000 (a monopoly where a single seller supplies the entire market). A HHI of > 1,000 is considered a concentrated market and > 2,000 highly concentrated [18]. HHI was calculated as the sum of the squares of each supplier’s market share each year. Market share by supplier was calculated as a percentage, by dividing the number of ITNs procured from each supplier in a specific year by the total number of nets shipped in the same year and multiplying by 100 [18]. ITN ‘contract manufacturers’ (manufacturers producing under license for another company) were classified under corresponding suppliers, according to their products and, where available, WHO manufacturer inspection reports [23]. The concentration ratio was calculated as the sum of the market share percentage held by the largest five suppliers in the industry each year. A concentration ratio between 0% to 50% is considered low, and generally indicates that the industry is competitive. An oligopoly exists when the top five firms in the market account for more than 60% of total market sales [13].

Sensitivity analysis was carried out to assess the impact on the results where the supplier was unknown.

##### Price determinants

Variability in the response variable *inflation adjusted unit price (ITN price, real)* was investigated using a generalised linear mixed model (GLMM) with a gamma distribution and log link function. Product brand name was a random effect, total ITNs shipped annually (continuous, in millions – an indicator of overall market size), HHI (continuous, in thousands), ITN area (continuous, in m^2^), ITN type (pyrethroid only, dual active ingredient (AI), or piperonyl butoxide (PBO), categorical), fibre type (polyethylene or polyester, categorical), inflation adjusted oil price ($/barrel) and geographical region (categorical) were treated as fixed effects. Global Fund introduced an ITN market shaping strategy in 2016, including the implementation of pooled procurement and reference pricing. Pooled procurement is a mechanism whereby the GF procures directly on behalf of multiple grantee countries to leverage economies of scale, and reference pricing refers to a published set of prices that serves to signpost expected prices to suppliers and consumers, in this case aiming to drive down the price. To account for these market shaping interventions, and to understand if price determinants were modulated by them, we included interaction terms between the above fixed effects and a binary *presence/absence* of the Global Fund Market Shaping Strategy (GFMSS) variable. We utilised a stepwise model selection process where non-significant predictor variables were eliminated to find the most parsimonious model that best explains the data.

### ITN retention and value for money considerations

#### Equivalent annual cost per person protected

Equivalent annual cost (EAC) was estimated for each country to account for different prices and retention times and allow like-for-like comparison of value for money (VfM). EAC was calculated following standard methods [24], combining data on median ITN retention time in 40 African countries [6] with price information from our database from 2017 to 2021 (see Online Resource for detail). We chose to use data on net retention rather than net durability because durability data is not available for all countries in our analysis.

$$EAC = {{P*r} \over {1 - {{(1 + r)}^{ - b}}}}$$

$$P = $$price

$$r = $$ discount rate

$$b = $$median retention time

Monte Carlo simulations were run in @Risk Software V 8.2 to simulate EAC per person protected and account for parameter uncertainty. In each country, one simulation was run for pyrethroid only ITNs, and another for PBO and Dual AI ITNs. Uncertain model inputs included median ITN retention time, ITN price, number of people protected per net, and the discount rate. A distribution was fitted to each uncertain input based on the available associated data (see Online Resource for input values and corresponding distributions).

#### Threshold price and premium for increasing retention

For purposes of our analysis, we defined baseline ITN retention time as the median retention time reported for each country from a recent analysis [25]. For each country, we compared value for money (VfM) of an ITN that is retained exactly 3 years (scenario 1) and 6 months more than the baseline retention time (scenario 2) to that of an ITN with baseline retention time. We explored the conditions under which an ITN that is retained for longer offers greater VfM than a net that is retained for the baseline retention time. ITN retention times and prices used in the analysis are detailed in the Online Resource.

The equation and simulations to estimate EAC described above were expanded to model the price P_l_ for a net that is retained for longer in each of the two scenarios, also referred to in this analysis as the threshold price, or willingness-to-pay [24]. Assuming procurers/countries are rational economic agents interested in maximising VfM, this price represents the maximum amount they would be willing to pay, in theory, for an alternative net that is retained for longer. In other words, it is the monetary value of additional longevity. The difference between this price and the baseline ITN price can be understood as a “premium” to be paid for increased retention, i.e. the marginal willingness-to-pay. The extended model is underpinned by the following equation [26]:

$$a = {{1 - {{\left( {1 + r} \right)}^{ - l}}} \over {1 - {{\left( {1 + r} \right)}^{ - b}}}}$$

$$a = $$relative price increase where the equivalent annual cost of a baseline net (EAC_b_) is equal to the equivalent annual cost of a net with increased retention (EAC_l_)

$$l = $$retention time of a longer retained net

$$b = $$retention time of a baseline net

$$r = $$discount rate

Based on *a*, the willingness-to-pay/threshold price (P_l_) of a longer retained net where EAC_b_ = EAC_l_ was calculated by multiplying the price of a baseline net by *a*. We then deducted the baseline price from (P_l_) to calculate the difference between the two prices, or the marginal willingness-to-pay for increased net retention to 3 years (scenario 1) or additional 6 months (scenario 2).

## Results

### Market analysis

**Fig. 1:**
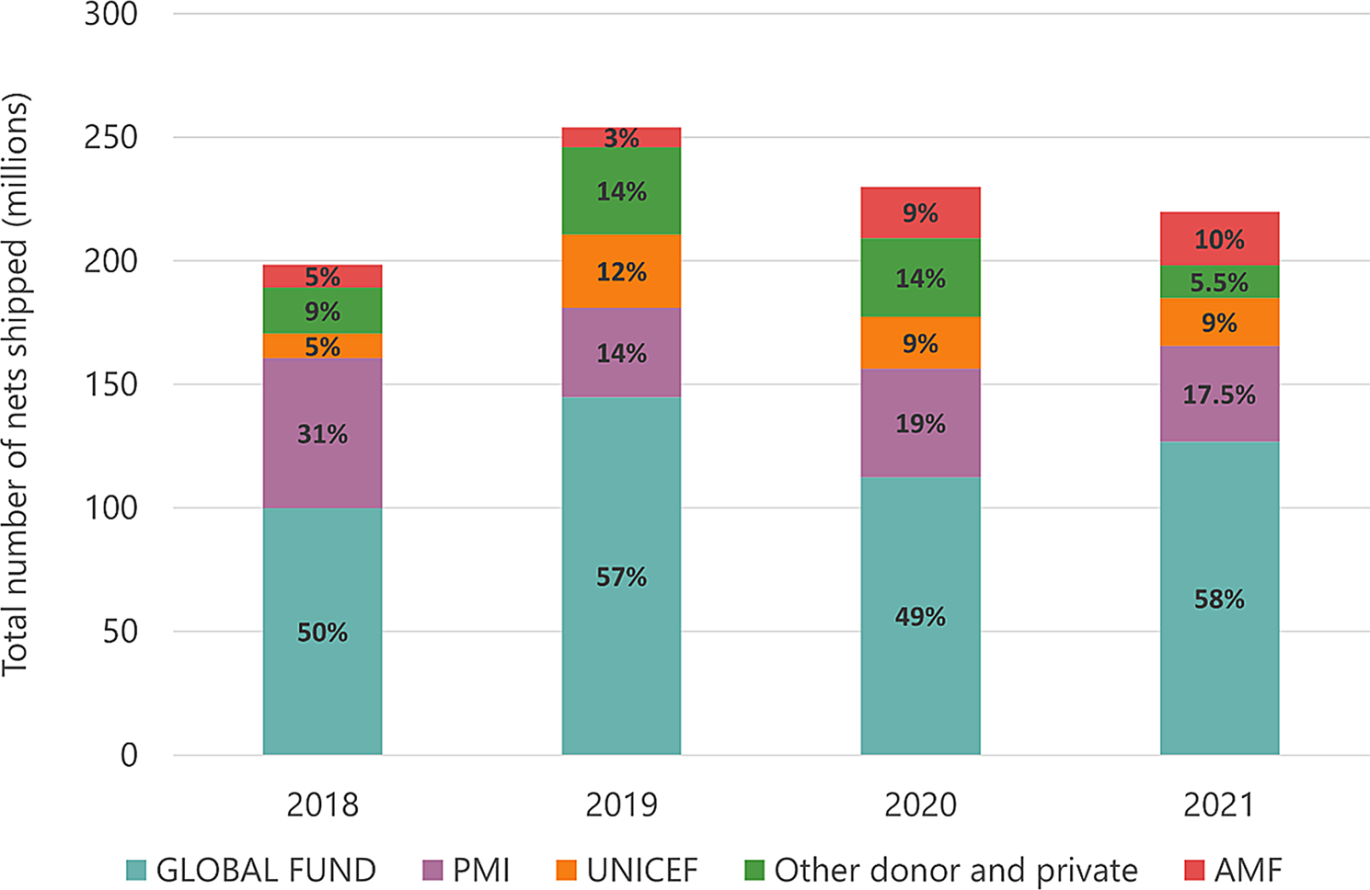
Global ITN shipments by major donor 2018–2021 data from the Alliance for malaria prevention. [21]. Abbreviations:PMI President’s malaria Initiative, UNICEF United Nations Children’s Fund, AMF against malaria Foundation

Our transaction data captured 69% of total nets shipped since 2004. Between 2018 and 2021, the ITN market was dominated by a handful of buyers, particularly Global Fund, PMI, UNICEF and the Against Malaria Foundation (Fig. 1). Global Fund funded over 50% of ITNs in all but one year. PMI has consistently been the second largest funder, accounting for 31% of ITNs bought in 2018, though this reduced to 18% by 2021. The combined share of ITNs supported by Global Fund and PMI reached 76% in 2021.

Our analysis shows that market segmentation in procurement and deployment may be warranted

**Fig. 2:**
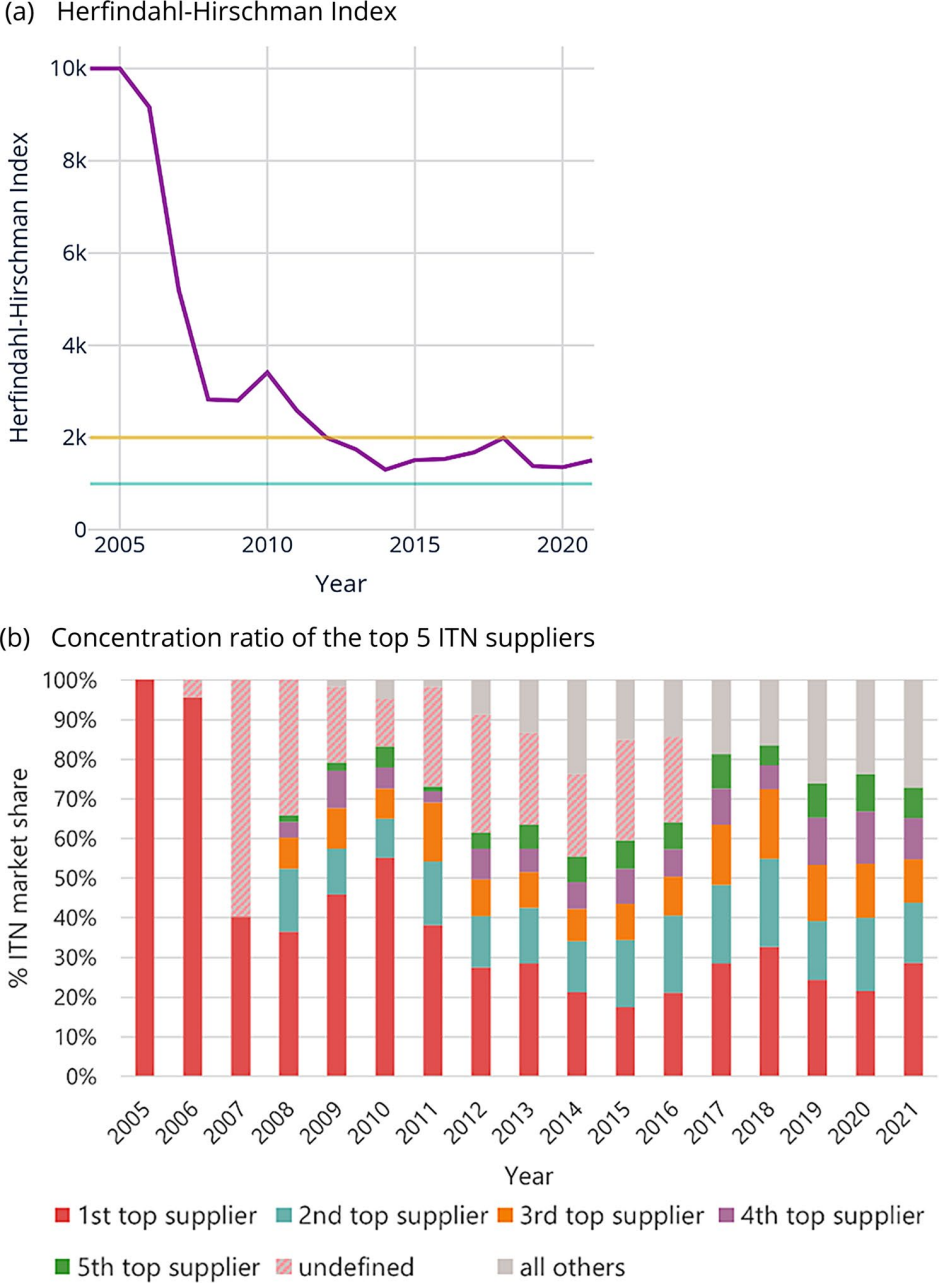
Measures of concentration in the ITN market Herfindahl-Hirschman index (**a**) and concentration ratio of the top 5 ITN suppliers (**b**) according to year of ITN delivery. HHI above the orange horizontal line in (**a**) represents a highly concentrated market, and HHI between the blue and orange horizontal lines represents a moderately concentrated market. Many ITN transactions had an undefined supplier (hatched area of bars in panel b), but prior to 2009, there were five or fewer suppliers so the concentration ratio of the top 5 ITN suppliers during this period was 100%. Abbreviations :ITN insecticide-treated bednet

The number of suppliers with ITN products approved by the WHO Pesticide Evaluation Scheme (WHOPES) or pre-qualified by WHO PQ-VCT rose from two in 2004 to fourteen in 2021. During the same period, the ITN market went from being highly concentrated (HHI = 10,000) to moderately concentrated (HHI = 1500) (Fig. 2 panel a). However, limited change occurred from 2013, and market concentration fluctuated between highly concentrated (HHI > 2000) and moderately concentrated (HHI = 1000 to 2000). The results are robust to varying assumptions on nets where the supplier is unknown (see Online Resource). The combined market share of the top five ITN suppliers (concentration ratio) has remained above 60% (except in 2014). The market share of the top supplier, which has varied over time, appears to show a downward trend, resulting in a more equal distribution between the top five suppliers in 2021 (Fig. 2 panel b).

### Price determinants

**Fig. 3:**
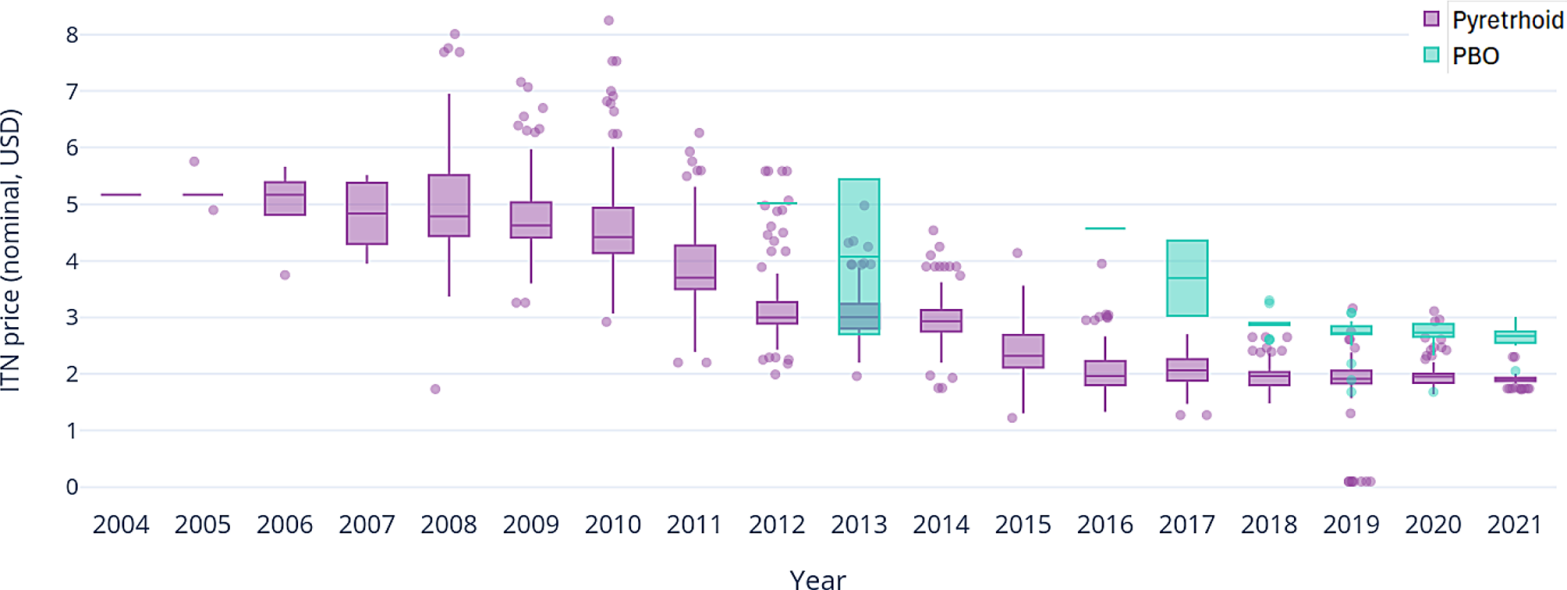
Unit prices of pyrethroid-only and PBO rectangular ITNs between 2004 and 2021 median price (thick line in boxes), first and third quartile (boxes), and range (whiskers). Circles indicate outliers (values greater or smaller than 1.5 times the inter-quartile range). Abbreviations: ITN insecticide-treated bednet, PBO piperonyl butoxide

The median price of pyrethroid-only ITNs has fallen since first coming to market, from $5.17 in 2004 to $1.90 in 2021. From 2017 to 2021, prices appeared to stabilise at around $1.90–$1.99 (Fig. 3). PBO net prices decreased from $4.67 in 2016 to $2.67 in 2021, resulting in a convergence of PBO and pyrethroid-only net prices. Price variability has reduced over time, but in-year differences in price remain, for both pyrethroid-only and PBO nets.

The most parsimonious regression model indicates that several variables were significant predictors of ITN price (Table 1).

**Table 1:** Model terms and their coefficients summary of estimated regression coefficients against real ITN price 2004–2021

	Coefficient	P-value	Lower 95% CI	Upper 95% CI
Intercept	−0.552	0.000	−0.657	−0.448
ITN type = Dual AI^a^	0.214	0.000	0.127	0.301
ITN type = PBO^a^	0.293	0.000	0.190	0.396
ITN area (m^2^)	0.042	0.000	0.038	0.046
ITNs shipped (millions)	−0.001	0.000	−0.001	−0.001
HHI (thousands)	0.253	0.000	0.242	0.263
GF market shaping present	−0.738	0.000	−0.938	−0.539
Oil price (tens $/bbl)*[GF market shaping present]	−0.042	0.000	−0.066	−0.019
Oil price (tens $/bbl)*[GF market shaping absent]	0.061	0.000	0.054	0.068
Region = Asia and the Pacific*[market shaping present]^b^	0.057	0.002	0.021	0.093
Region = Eastern Europe and Central Asia*[market shaping present]^b^	−0.015	0.783	−0.126	0.095
Region = Latin America and the Caribbean*[market shaping present]^b^	0.043	0.442	−0.067	0.153
Region = Middle East and North Africa*[market shaping present]^b^	−0.018	0.930	−0.422	0.386
Region = Asia and the Pacific*[market shaping absent]^b^	−0.065	0.000	−0.087	−0.043
Region = Eastern Europe and Central Asia*[market shaping absent]^b^	−0.005	0.855	−0.056	0.046
Region = Latin America and the Caribbean*[market shaping absent]^b^	0.088	0.000	0.046	0.130
Region = Middle East and North Africa*[market shaping absent]^b^	0.043	0.299	−0.038	0.123
ITNs shipped (millions)*[market shaping present]^c^	0.003	0.000	0.002	0.004

^a^Reference category pyrethroid only ITN

^b^Reference category sub-Saharan Africa

^c^Reference category market shaping absent

Dual AI Dual Active Ingredients, GF Global Fund, ITN Insecticide-treated Bednet, PBO Piperonyl Butoxide

Time period being after GFMSS commencement in 2016 was associated with a 52% lower price (exp(−0.738) = 0.478, *p* < 0.001, 95% CI 0.391–0.584). Net type, being PBO or dual AI, was associated with a 34% (exp(0.293) = 1.34, *p* < 0.001, 95% CI 1.21–1.49) and 24% (exp(0.214) = 1.24, *p* < 0.001, 95% CI 1.14–1.35) higher price, respectively, than pyrethroid-only ITNs. Every 1000 additional HHI points were associated with ITN price increase of 29% (exp(0.253) = 1.29, *p* < 0.001, 95% CI 1.28–1.30), and each additional square metre of fabric (i.e. larger net size) was associated with a 4.3% increase in expected price (exp(0.042) = 1.043, *p* < 0.001, 95% CI 1.038–1.046). The total number of nets shipped annually (proxy for market size) was associated with a 0.1% price reduction for every additional million nets shipped before GFMSS (exp(−0.001) = 0.999, *p* < 0.001, 95% CI 0.999–0.999) and a 0.3% price increase afterwards (exp(0.003) = 1.003, *p* < 0.001, 95% CI 1.002–1.004). Fabric type did not explain any variation in ITN price, so was excluded from the final model.

Price reductions associated with GFMSS also interacted with geographical location where nets were being shipped and, separately, oil prices. Net prices in Asia and the Pacific were associated with a 6% lower before (exp(−0.065) = 0.937, *p* < 0.001, 95% CI 0.916–0.958) and 6% higher after GFMSS implementation (exp(0.057) = 1.06, *p* = 0.002, 95% CI 1.02–1.10), relative to net prices in Sub-Saharan Africa. For every ten dollar per barrel increase in oil price, net prices rose by 6% before GFMSS (exp(0.061) = 1.06, *p* < 0.001, 95% CI 1.06–1.07) and fell by 4% after its introduction (exp(−0.040) = 0.96, *p* < 0.001, 95% CI 0.94–0.98).

### ITN retention and value for money considerations

#### Equivalent annual cost per person protected

**Fig. 4:**
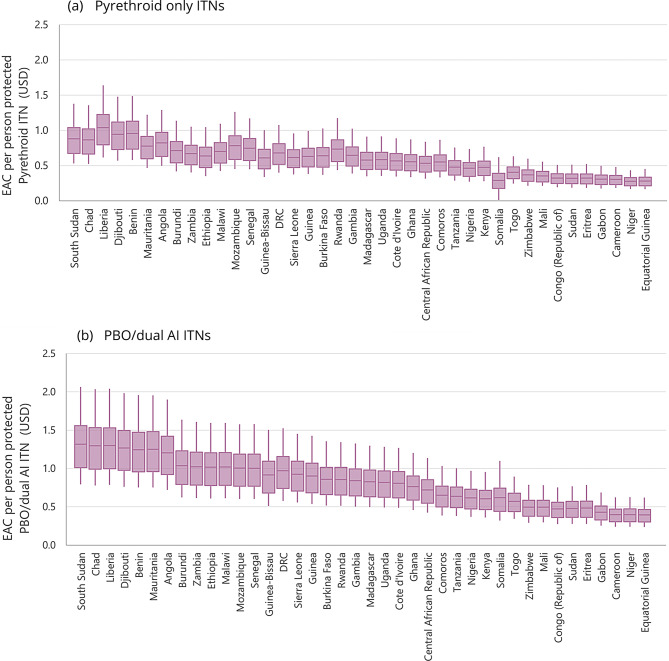
Simulated EAC per person protected by an ITN simulation results for EAC per person protected are displayed by country for pyrethroid-only (**a**) and PBO/dual AI ITNs (**b**). Countries are displayed along the horizontal axis and ranked according to estimated median net retention time (low to high). Mean simulated values (thick line in boxes), first and third quartile (boxes), and 90% CI (whiskers). Abbreviations: dual AI dual active ingredients, EAC equivalent annual cost, ITN insecticide-treated bednet, PBO piperonyl butoxide

EAC per person protected by an ITN ranges from $0.30–$0.97 (pyrethroid-only) and $0.35-$1.36 (PBO and dual AI). There is an inverse relationship between EAC per person protected and median retention time (Fig. 4), with countries exhibiting longer retention times consistently achieving lower annualised costs per person protected. This relationship is observed for both pyrethroid-only and PBO/dual AI ITNs, indicating that durability and retention are key drivers of value irrespective of insecticide class.

Although PBO and dual AI ITNs generally have higher EACs, reflecting higher unit prices, there is substantial overlap in the EAC distributions between ITN types, particularly in countries with longer retention times. This suggests that higher-priced ITNs can offer similar or lower costs per person protected when they last longer. In contrast, short retention times lead to high EACs for all ITN types, showing that poor durability reduces value regardless of insecticide type.

The uncertainty in the simulated values is due to the wide range assumed for the number of people protected per net (from 1 to 4 people), with retention time, price and choice of discount rate also impacting uncertainty (Online Resource). Despite this uncertainty, the downward trend in EAC with increasing retention time is consistent across countries, underscoring the potential efficiency gains from improving ITN physical durability and useful lifespan. These findings support the incorporation of durability and realised lifespan into procurement decisions, rather than reliance on unit price alone.

#### Threshold price and premium for increasing retention

**Fig. 5:**
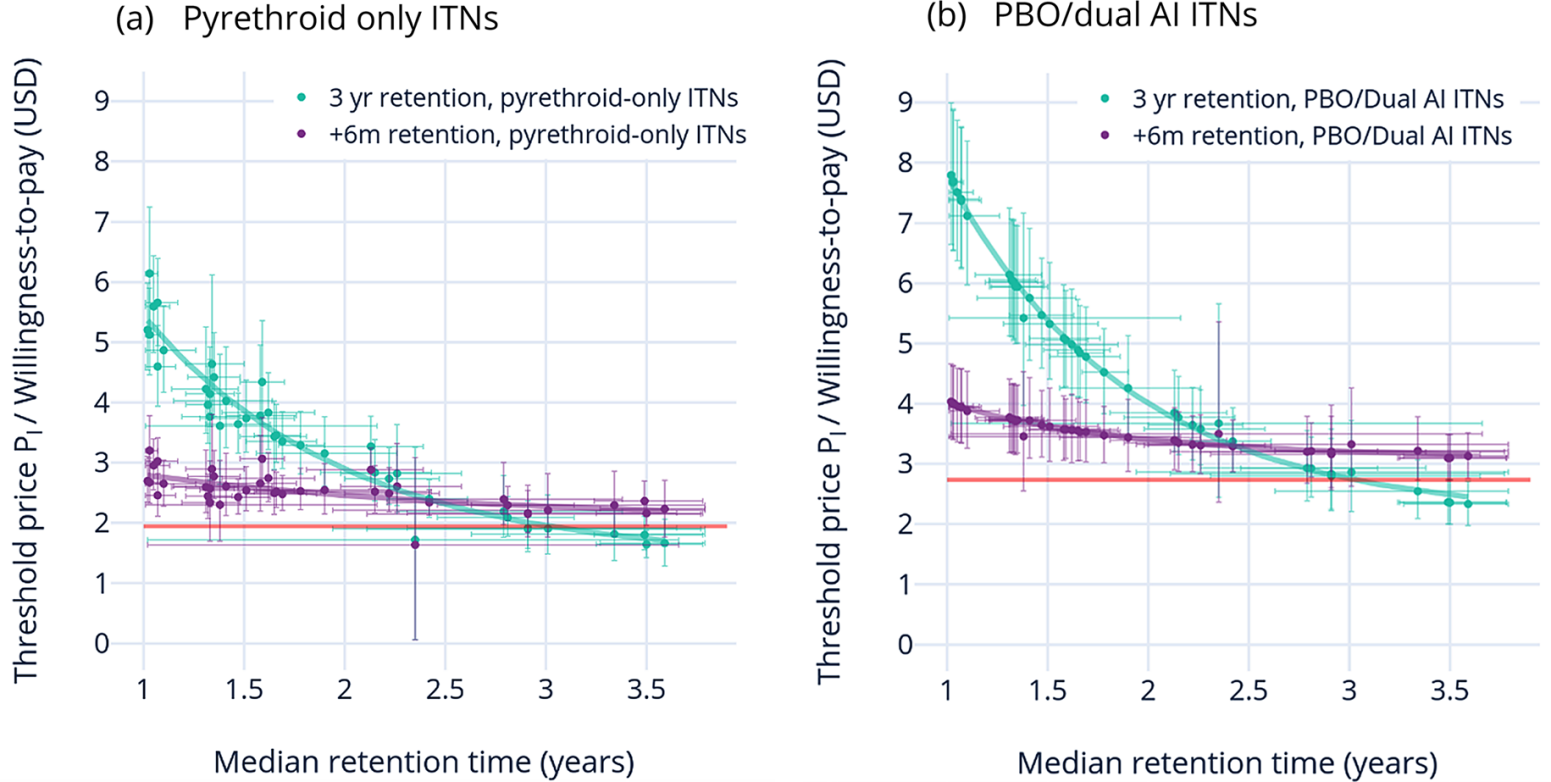
Threshold price P_l_ (willingness-to-pay) for ITNs with increased retention time mean simulated threshold price (±90% CI) of an ITN with increased retention is plotted against baseline median retention time estimates (±90% CI) from Bertozzi-Villa et al. [6] each dot represents the simulated value from a single country. Green dots (and associated green exponential line of best fit) represent scenario 1 where retention time is increased to 3 years, and purple dots (and associated purple exponential line of best fit) represent scenario 2 where retention time is increased by 6 months. Panel a shows threshold price of a pyrethroid-only ITN, and panel b shows threshold price of a PBO/dual AI ITN. Red horizontal lines represent the mean simulated price of a pyrethroid-only (in a) or PBO and dual AI (in b) ITN

Modelled threshold price P_l_ (or willingness-to-pay) for an ITN with longer retention decreases exponentially as baseline median ITN retention times increase for both pyrethroid-only (Fig. 5a) and next generation ITNs (Fig. 5b). Threshold price is generally lower for pyrethroid-only nets because of their lower baseline price.

P_l_ starts higher and decreases with a steeper slope in scenario 1 (increase in retention to 3 years) than in scenario 2 (increase in retention by 6 months) because the difference between baseline and increased retention time is much higher where baseline retention values are close to 1, and eventually reach negative values when baseline retention is already higher than 3 years.

The difference between P_l_ and baseline price, or the premium for longer retention (marginal willingness-to-pay), decreases as baseline ITN retention time increases. In scenario 1, a negative premium would in theory be generated where baseline retention is already above 3 years.

## Discussion

Our analysis demonstrates several important ITN market failures relative to perfect competition, including some potential unintended consequences of market shaping interventions. We found an oligopsony market characterised by a small number of the same buyers who accounted for a large share of ITN purchases over the period studied. Similarly, we found evidence of oligopoly, with a small number of suppliers, accounting for a large proportion of the market. While market concentration declined over the period studied, the market remained concentrated between five major suppliers.

Despite the seemingly uncompetitive market, the price of both pyrethroid only and PBO ITNs fell and converged over the period studied. Dual AI, PBO ITNs and larger ITNs were associated with a higher price. ITN price was negatively associated with the presence/absence of the GFMSS, and positively associated with market concentration (measured through HHI). The implementation of the GFMSS in 2016 [27] was likely successful in achieving one of its objectives to improve ITN affordability. However, the steep drop and subsequent levelling of HHI occurred at roughly the same time as the implementation of the GFMSS, so it is difficult to disentangle their individual effects on price. Similarly, time-limited, supply-side subsidies to promote access to dual-AI ITNs may also have influenced market and price dynamics. Interestingly ITN and oil prices were positively associated in the absence of GFMSS and negatively associated after GFMSS was introduced, suggesting that the market shaping strategy interfered with the ability of ITN producers to pass on increases in raw material costs. It is also possible that early procurement practices accompanying the introduction of the GFMSS—such as advance purchasing or large-volume contracting at fixed prices—temporarily dampened the transmission of higher raw material costs, including oil prices, to ITN end prices.

Outside of our analysis, other market failures exist in the ITN market which may also affect the efficiency of this market. Information about products is not readily accessible to buyers and users, and there is unequal (asymmetrical) and limited (imperfect) access to information. Imperfect information creates risk that buyers will lower their willingness-to-pay and thus drive more expensive, higher-performing products out of the market [28]. WHO product registration and national regulatory standards can, to some extent, overcome these information problems, by giving buyers and users confidence that products meet pre-defined standards. However, there is a risk that these regulations create barriers to entry for new suppliers and innovative products, potentially stifling innovation and keeping prices higher than they might be under greater competition. A deeper understanding and careful market monitoring is necessary to ensure the appropriate incentives are in place to catalyse innovation while simultaneously achieving other desired outcomes. As described by WHO, fostering innovation may require allowing manufacturers to initially capture some economic surplus, provided this is balanced with measures to ensure equitable access and long-term market sustainability [29, 30].

One promising development for improving information on ITN durability is resistance to damage (RD), a performance metric designed to capture ITN’s ability to withstand mechanical stress. RD has recently been developed as a key performance metric to evaluate the durability of the ITNs. The RD score is a composite score consisting of several measures of an ITN’s ability to withstand mechanical forces [31]. Evidence suggests that ITNs with better RD scores are retained for longer [32], meaning that this metric could be useful in comparing the predicted relative functional lives of multiple products before they are purchased. Value-based procurement provides a mechanism to translate RD scores into practical purchasing decisions. Procurers, particularly those with significant market leverage such as major donors, can use the RD metric to establish willingness-to-pay for longer-lasting nets, ensuring that procurement choices reward durability while promoting equitable access and maintaining cost-effectiveness. The RD score was recently refined with new weights assigned to each measurement, making an even better predictor of physical durability [33]. In 2022, the WHO Pre-qualification Team for Vector Control (WHO PQT-VC) updated its guidelines to include pre-market assessment of measurements included in the RD score [34]. This update represents an important first step toward ensuring more durable and effective malaria control tools, and it is crucial that countries and procurers effectively apply this new information when selecting products.

To put the RD metric into practice, procurers should look beyond unit price and consider the cost per year of effective life, combining RD scores with context-specific retention times. This approach allows products to be compared based on predicted durability and value for money, rather than just upfront cost. Manufacturers would need to report RD scores transparently and provide post-market surveillance data to support these calculations. Using RD-informed effective life in purchasing decisions would give agencies like the Global Fund or national malaria programmes the leverage to incentivize the development and distribution of longer-lasting ITNs, aligning procurement with both public health impact and cost-effectiveness.

We used real ITN price data and modelled estimates of ITN retention times across sub-Saharan Africa to estimate the equivalent annual cost (EAC) of existing products and willingness-to-pay for hypothetical longer-retained ITNs. We found that the value for money offered by an ITN is context-specific and largely depends on existing ITN retention times, though price and other modelling assumptions also influence the results. Quantifying the value of a longer retained ITN, as done in this study, could help national malaria programmes (NMP) and partners to make context-specific value-based decisions on ITN products. In addition to retention and efficacy, environmental impacts—including production emissions, deployment waste, and disposal considerations—should be incorporated into value-based decision-making. Accounting for these factors alongside physical durability and chemical efficacy can help NMPs and donors make procurement choices that maximise both public health and ecological benefit. While our analysis focuses primarily on physical durability, this represents only one component of ITN value; a more comprehensive evaluation—including bioefficacy, environmental impact, and broader societal benefits—would provide a fuller understanding of the true value of different ITN products.

It is also important to recognise that net retention is influenced not only by physical durability but also by the frequency and efficiency of ITN distribution. In contexts where nets are over-distributed to easy-to-reach populations, functional nets may be replaced before they wear out, artificially shortening observed retention times. Without improvements in distribution efficiency, this could reduce the population-level returns on investing in more durable nets and, in turn, limit willingness to pay for additional durability.

The case for a meaningful and practical value metric for ITNs is strengthened as global net procurement shifts towards chemically innovative and more expensive products [7, 12] however, a key challenge is that the ITN market suffers from the principal-agent problem where one party (the agent) acts on behalf of another (the principal), without perfectly aligned incentives. The problem arises if agents prioritise their own objectives over the principals, leading to sub-optimal outcomes for the principal. In ITN markets, the principals, ITN consumers (end users) do not purchase them directly, hence their knowledge about the real useful life of nets, may not be considered in purchasing decisions made by agents (donors and governments) with limited budgets who may be incentivised to purchase cheaper nets and achieve short-term coverage targets. This is compounded by lack of data (and perhaps limited capacity) to assess true value for money in different contexts. Together these factors make value-based procurement very challenging. Building on recently published information about products [10] and recommendations on geographic targeting [35], the present analysis provides a basis to develop a practical tool allowing countries to better evaluate and justify their ITN product choices to donor agencies, potentially providing evidence to mitigate the principal-agent problem.

Our analysis shows that market segmentation in procurement and deployment may be warranted, i.e. to place more robust ITNs in areas with the shortest current retention times, particularly given severely constrained budgets and competing demands that are ever increasing. This approach is based on the assumption that historical differences in retention times largely reflect contextual or “background” factors rather than inherent differences in product robustness. Advances in techniques to measure physical durability of ITNs [36] lay the foundations to incentivise development of higher performing products, but the financial implications and the potential impact on the ITN market are unclear.

While this study focuses on retention as a key driver of value, it is important to recognise that the physical integrity of ITNs contributes directly to their protective effectiveness against mosquito exposure and malaria infection. Field studies show that ITNs develop holes over time, which weakens their ability to act as a physical barrier and reduces protection even when nets continue to be used [37]. Quantitative analyses indicate that physical damage contributes to measurable losses in effectiveness against malaria transmission, highlighting that durability is a meaningful component of overall ITN impact [38]. These findings support the view that physical damage and retention are not just markers of attrition but are causally linked to reduced protective performance, underscoring the importance of including durability metrics alongside chemical bioefficacy when assessing ITN value.

One limitation of our analysis is that the modelled estimates of ITN retention times implicitly assume that retained nets remain biologically active, which may not be the case. In practice, ITN effectiveness depends on the interaction between its physical integrity and the persistence of insecticidal bioefficacy, both of which decline over time.

Future work could therefore extend this framework by incorporating bioefficacy, for example by defining an effective life based on the period during which a retained net continues to meet a specified bioassay mortality threshold. This would allow value assessments and ultimately procurement decisions, to better reflect the combined contribution of physical durability and insecticidal performance over time.

We did not include a budget constraint or consider ITN distribution costs in our analyses. In reality, where financial resources are limited, switching to a higher cost (albeit longer retained) ITN may not be affordable, and result in a coverage trade-off, potentially offsetting gains made. The trade-off between investing additional resources in a longer retained ITN, versus increasing the frequency of campaign cycles (ie to two-year campaign cycles) was also not explored. Distribution as well as other costs should be captured in further work.

## Conclusion

While significant progress has been made in improving the affordability, accessibility, and distribution of ITNs over the past decades, these advancements are at risk of stagnating if the overall health of the ITN market is not addressed. From 2004 to 2021, the market shifted from being highly concentrated to moderately concentrated, and despite improvements in availability, quality has not substantially differentiated between ITN products. This suggests that while affordability and access have improved, innovation in quality may be lagging, which could hinder further progress in malaria prevention

Findings from previous studies have suggested that longer-retained nets would likely be more cost-effective, but have not gone as far as estimating a price premium which reflects the economic value of increased ITN retention. There is a risk that if ITNs are not performing as expected and countries begin two-year ITN campaign cycles, the false economy of not investing in better performing products will severely affect countries’ ability to maintain effective ITN coverage and have a serious impact on global malaria elimination goals. A move to value-based procurement is therefore critical.

Ideally, context specific functional ITN survival data obtained through durability studies, combined with data on RD scores for different products should be used to predict ITN value in different settings, as well as inform replenishment and future campaign cycles. Results of this study could be used by malaria control programmes to justify selection of higher-priced higher-value nets if they had information on ITN retention and durability of specific products in their own context. This could incentivise manufacturers to improve ITN strength and durability, translating into longer retention times, improved efficiency and public health gain.

## Electronic supplementary material

Below is the link to the electronic supplementary material.

Supplementary Material 1

## Data Availability

The data used in this analysis is available from the Global Fund Price Quantity reporting database. PMI data was obtained directly from PMI and any readers wishing to obtain these data should contact PMI directly.
