# Supplementary material for: Analysis of insecticide-treated bednet market dynamics between 2004–2021 and monetary value of additional bednet longevity
Source: Cost Eff Resour Alloc. 2026 Feb 25;24:41. doi: 10.1186/s12962-026-00725-0 (PMC12969863; doi:10.1186/s12962-026-00725-0)
Supplement: Supplementary file 1 — Supplementary Material 1 [file 12962_2026_725_MOESM1_ESM.pdf]

Supplementary Information – Analysis of Insecticide-Treated Bednet market dynamics between 2004-2021 and monetary value of additional bednet longevity

Journal: Applied Health Economics and Health Policy

Amanda McCoy[1*]

Edward Thomsen[2**]

Angus Spiers[2***]

Eve Worrall[1]

[1]Liverpool School of Tropical Medicine, Clinical Sciences Department, Pembroke Pl, Liverpool L3 5QA, United Kingdom, +44 151 705 3759

[2]Innovation to Impact, Liverpool School of Tropical Medicine, Pembroke Pl, Liverpool L3 5QA, United Kingdom, +44 151 705 3759

*Corresponding author: Amanda.McCoy@lstmed.ac.uk, +44 151 705 3759

**Current affiliation: Malaria Elimination Initiative, Institute for Global Health Sciences, University of California, San Francisco, USA

***Current affiliation: Oxitec Ltd, 71 Innovation Dr, Milton Park, Abingdon, OX14 4RQ, UK

Contents

GF PQR and PMI data cleaning and enhancing

Table S1

Macro-economic indicators

Monte Carlo simulation methods

Table S2

Table S3

HHI Sensitivity analysis

Figure F1

Price determinants

Figure F2

EAC sensitivity analysis

Figure F3

GF PQR and PMI data cleaning and enhancing

The final download of Global Fund Price and Quality Reporting (GF-PQR) database took place on 10.03.22, and data received by the President's Malaria Initiative (PMI) on 27.01.22 was used. A total of 3669 transaction lines were included in the analysis, of which 2338 from GF-PQR. Transactions flagged as operational research by PMI were not included in the final analysis but were kept in the database for information purposes. Transaction lines with no quantity or price data were also included in the database for information, but could not be used in the analysis. While all GF prices include accessories (hooks, strings, bag), this may or may not be the case for PMI prices, and it is not possible to make a distinction. As they represent less than 10% of LLIN cost, this issue was not believed to affect the overall results.

The combined database was prepared following three main stages. Firstly the GF PQR database, available from Workbook: Price & Quality Reporting Transaction Summary (theglobalfund.org) was downloaded and cleaned. Data was selected from drop down menus to include all products within "bednet/IRS" category and within those all ITN products (excluding non-treated nets). In order to ensure the cleaning process was easily reproducible and errors could be traced if necessary, the cleaning was carried over of a series of worksheets, and no lines were deleted from the data downloaded originally. This approach also helps to highlight transactions with incomplete information at the time of download (e.g. quarantined information). Where necessary and possible, minor corrections were made directly into existing columns. All manual alterations can be identified by juxtaposing columns from the clean dataset with original data. Two types of minor revisions were carried out manually. The first consisted in making data entries consistent with each other (consistency check), e.g. country, product, manufacturer names, etc. The second consisted in revisions to data entries where errors were spotted (e.g. through filtering data) and that could be performed with certainty, including:

- net shape based on given dimensions
- product name wherever possible (including where the product named as "LLIN" but the actual name was clear)
- the order in which net dimensions were given
- LLIN unit price, where there was a clear typo (this was done only once for a price of $211 which should have been $2.11, as per price for transaction above and below)
- Product entered as an ITN but description as IRS-in this case the transaction line was kept, but the information relating to IRS removed

In a second instance, once the GF PQR data had been cleaned, PMI data was added and formatted as necessary, to ensure consistency. The third stage consisted of adding variables considered useful for the analysis and completing final checks. A number of new categories were created, in order to facilitate and enhance the analysis. In some instances, categories existing in PMI data had to be created for GF -PQR data and vice versa. The data was further checked column by column, in order to ensure consistency across entries and additional spot checks were also carried out, particularly where large quantities of nets were involved. Checks were made to ensure no missing quantity or price data was entered as 0, to avoid skewing the statistical analysis.

S1 Additional database categories

Name of additional column	Information source/type	Column content
Caveat	Flags transactions representing operational research and/or where delivery date is estimated	Combined OR flag with note of estimated delivery
Product	ITN product name	For PMI data, product names were entered based on manufacturer and insecticide (no information available prior to 2017)
Region	Classification of countries by Global Fund (investment) region	

Procurement channel (i.e.	Simplified procurement channel	Data grouped into 3 categories:<br>-Pooled Procurement Mechanism (PPM)<br>-Direct from Manufacturer (Mfg)<br>-Other (includes all development partners and private procurement agents)
Supplier	Main product supplier/parent company (may be the same as manufacturer)	Simplified from "manufacturer" variable, in order to group all contract manufacturers/manufacturing facilities under main supplier<br>Where manufacturer is not recognized as WHO approved or as a contract manufacturer of approved supplier, classed as "other"<br><br>For PMI data, this is the same as "manufacturer", though no information on the latter is available prior to 2017
LLIN type	Pyrethroid, PBO or dual AI	Data added for PQR data
Material	Polyester, polyethylene, polypropylene (one off) or combination	Data added for PQR data
Height >, = or < than 170cm	Describes whether net height is greater, equal or less than 170cm	Data added. Based on existing measurements
Net area (in m2)	Total net area	Calculated for rectangular and conical nets according to corresponding formulae
Net area (large or small)	Describes whether total net area is considered large or small	Any area equal to above 14.5m2 is considered large, and small for an area below 14.5m2
Number of LLIN suppliers	Number of LLIN suppliers offering ITN products pre-qualified by WHO in PO year	Data added based on review of WHOPES reports, WHO-PQT database and UNICEF procurement data
Number of LLIN products	Number of LLIN products pre-qualified by WHO in the PO year	Data added based on review of WHOPES reports, WHO-PQT database and UNICEF procurement data
Total number of LLINs	Total of LLINs shipped in the PO Year	Data added based on NMP
LLIN Unit cost (nominal, adjusted for freight)	ITN cost adjusted for freight	Where freight cost was noted as "embedded in the price", cost was adjusted down by 15%, representing a revised figure from GF indicative freight costs given for 2021, in order to reflect a more accurate image of pre-covid values[1].
LLIN Unit cost (real)	ITN cost adjusted for inflation	Price index was created with 2003 as the base year, in order to facilitate cost comparisons over the years[2]. Projections were used for 2021, as finalized values were not available.
LLIN Unit cost (real, adjusted for freight)	Real ITN cost adjusted for freight cost	As above

PO year	Year of purchase	Converted PO dates to year in order to enable comparison with net mapping project data and other variables such as number of approved manufacturers, oil price, etc.
Oil Price	Price of oil in PO year	Based on World Bank Data[3]
Year of delivery	Year of delivery	Converted all dates to year to facilitate comparison with MAP.
Lead time	Time between PO date and actual delivery	Data added using appropriate formula Calculation was not possible where inconsistencies existed between the two dates
Herfindahl-Hirschman Index (HHI)	Measure of market concentration	Calculated as per given formula[4]

Macro-economic indicators

The average price of oil (across all types) was obtained from the World Bank[3]. All costs are in USD and are adjusted for inflation, also using World Bank data[2].

Monte Carlo simulation methods

Monte Carlo simulations were run in @Risk Software version 8.2 to simulate EAC per person protected in each country for standard and PBO/dual AI ITNs (model output) in the 40 countries where retention estimates were available. Uncertain model inputs included median net retention time, ITN price, number of people protected per net and a discount rate.

ITN retention data from 2000-2020 across the 40 highest-burden African countries was obtained from a study published in 2021[5]. For purposes of the simulation, median and 95% Confidence Interval (CI) values were used (as supplied by the original study) to fit a triangle distribution to this data, as shown in table S2.

Price data from 2017-2021 from our combined GF-PQR and PMI database were used for all countries in order to capture as many transactions as possible while avoiding those from earlier years when prices were notably higher. Wherever possible, @ Risk Software version V 8.2 was used to fit a distribution to each individual set of prices per country. Median and 95%CI values are displayed in table S3 below, alongside the corresponding fitted distribution and key parameters. The @Risk fitted distribution had to be truncated in one instance where the distribution returned a negative value for 95%CI low. This occurred in Somalia where a set of $0.1 unit cost data points were recorded and deliberately kept in the data, as there was no indication of an entry error having been made. Where the data range was insufficient to fit a distribution (four or less price points), values of + or – 15% from the median were used to generate a 95% CI, and a triangle distribution was assumed for the simulation.

Due to a small number of observations and a small difference in the price premium paid for dual AI and PBO nets relative to pyrethroid-only ITNs, data for PBO and dual AI nets were grouped in this analysis. For the ITN price data, prices for pyrethroid-only (standard) nets (conical and rectangular) were disaggregated by country wherever data were available, while those for PBO and dual AI nets were aggregated across the entire dataset. Where no price information on standard nets was available for a particular country, the distribution fitted to the full set of prices for pyrethroid-only nets 2017-2021 was used (this only occurred in two cases out of forty, see table S3).

Drawing from existing literature[6], the number of people sleeping under a net was assumed to vary from 1 to 4, with a mean of 1.8. A standard discount rate of 3% was applied[7], and assumed to vary from 2% to 4%. A triangle distribution was assumed in the sensitivity analysis for both the number of people protected per net and the discount rate.

In total, 80 simulations were run (for pyrethroid-only and PBO and dual AI ITNs in 40 countries) with 100,000 iterations per simulation.

Table S2. Retention data[5] used in simulation of EAC per person protected and price threshold for "increased retention" for standard and PBO/dual AI ITNs

Country	Median	95% CI Low	95% CI High
South Sudan	1.02	1.01	1.04
Chad	1.03	1.01	1.08
Liberia	1.03	1.01	1.07
Djibouti	1.05	1.01	1.13
Benin	1.07	1.01	1.17
Mauritania	1.07	1.01	1.16
Angola	1.1	1.01	1.26
Burundi	1.31	1.14	1.47
Zambia	1.32	1.21	1.44
Ethiopia	1.33	1.19	1.48
Malawi	1.33	1.21	1.45
Mozambique	1.34	1.21	1.5
Senegal	1.35	1.22	1.48
Guinea-Bissau	1.38	1.01	2.16
DRC	1.41	1.15	1.64
Sierra Leone	1.47	1.31	1.63
Guinea	1.51	1.28	1.75
Burkina Faso	1.58	1.41	1.76
Rwanda	1.59	1.48	1.7
Gambia	1.62	1.39	1.85
Madagascar	1.65	1.48	1.81
Uganda	1.66	1.55	1.78
Cote d'Ivoire	1.69	1.51	1.86
Ghana	1.78	1.67	1.9
Central African Republic	1.9	1.56	2.26
Comoros	2.13	1.81	2.39
Tanzania	2.15	1.88	2.43
Nigeria	2.22	2	2.47
Kenya	2.26	1.98	2.58
Somalia	2.35	1.02	3.66
Togo	2.42	2.21	2.61
Zimbabwe	2.79	2.26	3.38
Mali	2.81	2.46	3.14
Congo (Republic of)	2.91	2.31	3.65
Sudan	2.91	2.11	3.77
Eritrea	3.01	1.94	3.79
Gabon	3.34	2.63	3.79
Cameroon	3.49	3.24	3.78
Niger	3.5	3.25	3.78
Equatorial Guinea	3.59	3.27	3.79

Table S3. Unit price data and distribution functions in simulation of EAC per person protected and price threshold for "increased retention" for standard and PBO/dual AI ITNs

Input category	Country	Median	95% CI low	95% CI high	Distribution Function (parameters)	Number of price points/notes
Standard ITN price	South Sudan	1.74	1.73	2.11	RiskPareto(θ=18.6,α=1.73)	6
	Chad	1.83	1.83	1.89	RiskPareto(θ=115.6,α=1.83)	5
	Liberia	2.25	1.89	2.48	RiskUniform(min=1.8, max=2.5)	7
	Djibouti	1.84	1.56	2.02	RiskTriangle (most likely, min, max)	2
	Benin	2.1	1.97	2.23	RiskLaplace (μ=2.1,σ=0.06)	10
	Mauritania	1.73	1.47	1.9	Assumed Triangle (most likely, min, max)	1
	Angola	1.87	1.7	2.04	RiskLaplace(μ=1.87,σ=0.08)	13
	Burundi	1.83	1.7	2.42	RiskPareto (θ=10.4,α=1.7)	14
	Zambia	1.76	1.54	1.94	RiskTriangle (most likely=1.95, min=1.46, max= 1.95)	25
	Ethiopia	1.76	1.25	2.18	RiskUniform (min=1.22, max=2.2)	75
	Malawi	1.9	1.78	1.98	RiskUniform (min=1.77, max=1.99)	11
	Mozambique	2.02	1.86	3.03	RiskPareto (θ=7.5, α=1.85)	35
	Senegal	1.98	1.86	2.32	RiskExtValue (alpha=1.98, beta=0.09)	20
	Guinea-Bissau	1.79	1.69	2.2	RiskPareto (θ=13.89,α=1.69)	5
	DRC	1.88	1.66	2.17	RiskUniform (min=1.64, max=2.19)	81
	Sierra Leone	1.92	1.63	2.1	Assumed Triangle (most likely, min, max)	4
	Guinea	1.86	1.85	2.12	RiskPareto (θ=27.52, α=1.85)	12
	Burkina Faso	1.97	1.78	2.85	RiskPareto (θ=7.73,α=1.77)	12
	Rwanda	2.3	2.13	3.03	RiskPareto (θ=10.33,α=2.12)	6
	Gambia	2.14	1.82	2.35	Assumed Triangle (most likely, min, max)	3
	Madagascar	1.95	1.66	2.18	RiskLogistic (α=1.92, β=0.07)	49

	Uganda	1.92	1.86	2.22	RiskPareto (θ=21.1,α=1.86)	18
	Cote d'Ivoire	1.85	1.85	2.11	RiskPareto (θ=27.77,α=1.85)	7
	Ghana	1.97	1.79	2.31	RiskExtValue (alpha=1.93, beta=0.1)	22
	Central African Republic	2.06	1.69	2.19	Assumed Triangle (most likely, min, max)	3
	Comoros	2.3	1.93	2.5	Assumed Triangle (most likely, min, max)	4
	Tanzania	2.06	1.86	2.37	RiskTriangle (most likely=1.85, min=1.85, max=2.47)	77
	Nigeria	1.96	1.94	2.67	RiskLognorm(μ=0.11, σ=0.44, Riskshift (1.94))	59
	Kenya	1.96	1.9	2.94	RiskPareto (θ=8.46, α=1.9)	26
	Somalia	1.88	0.1	1.98	RiskPert (most likely=2, min=0.1 max=2)	27/distribution truncated at 0.1
	Togo	1.96	1.81	2.03	RiskExtValue (alpha=1.97,beta=0.04)	6
	Zimbabwe	1.95	1.85	2.66	RiskPareto (θ=9.99, α=1.84)	20
	Mali	1.93	1.8	2.2	RiskExtValue (alpha=1.91, beta=0.08)	22
	Congo (Republic of)	1.94	1.64	2.13	Assumed Triangle (most likely, min, max)	2
	Sudan	1.85	1.78	1.99	RiskPareto (θ=33.79, α=1.78)	5
	Eritrea	1.85	1.57	2.04	Assumed Triangle (most likely, min, max)	3
	Gabon	1.95	1.43	2.47	RiskLaplace (μ=1.95, σ=0.24)	No country price points available, full set of standard ITN used
	Cameroon	2.03	1.96	2.46	RiskPareto (θ=16.37, α=1.96)	10
	Niger	1.92	1.77	2.05	RiskUniform (min=1.76, max=2.05)	14
	Equatorial Guinea	1.95	1.43	2.47	RiskLaplace (μ=1.95, σ=0.24)	No country price points available, full set of standard ITN used
PBO and dual AI ITNs	All countries	2.74	2.39	3.08	RiskNormal (μ=2.7, σ=0.18)	Full set PBO and dual AI

HHI sensitivity analysis

Given the volume of nets for which there is no supplier information, a sensitivity analysis was carried out on HHI values, varying the assumptions on how to distribute nets with unknown supplier as follows:

Variation 1: All nets from unknown suppliers from 2008 were split across suppliers already recorded in the combined database, according to their existing annual percentage share of ITNs. Prior to 2008, all nets with unknown supplier were split equally between the only supplier already included in the combined database and a second supplier whose ITN product was pre-qualified by WHO but does not appear in the combined database until 2008.

Variation 2: All nets from unknown suppliers in a given year were split equally across all suppliers known to have ITN products pre-qualified by WHO and in circulation on the market during that particular year.

Variation 3: All nets from unknown suppliers were assigned to the largest supplier in any given year (based on net volume).

Figure F1 below shows the result of the sensitivity analysis on HHI values under different scenarios. Market concentration is lower from 2004-2007 where two, rather than one supplier were included in the analysis. If all nets from unknown suppliers were allocated to the largest supplier (variation 3), market concentration increases for all years.

Figure F1. Sensitivity analysis results for HHI in the ITN market 2004-2021

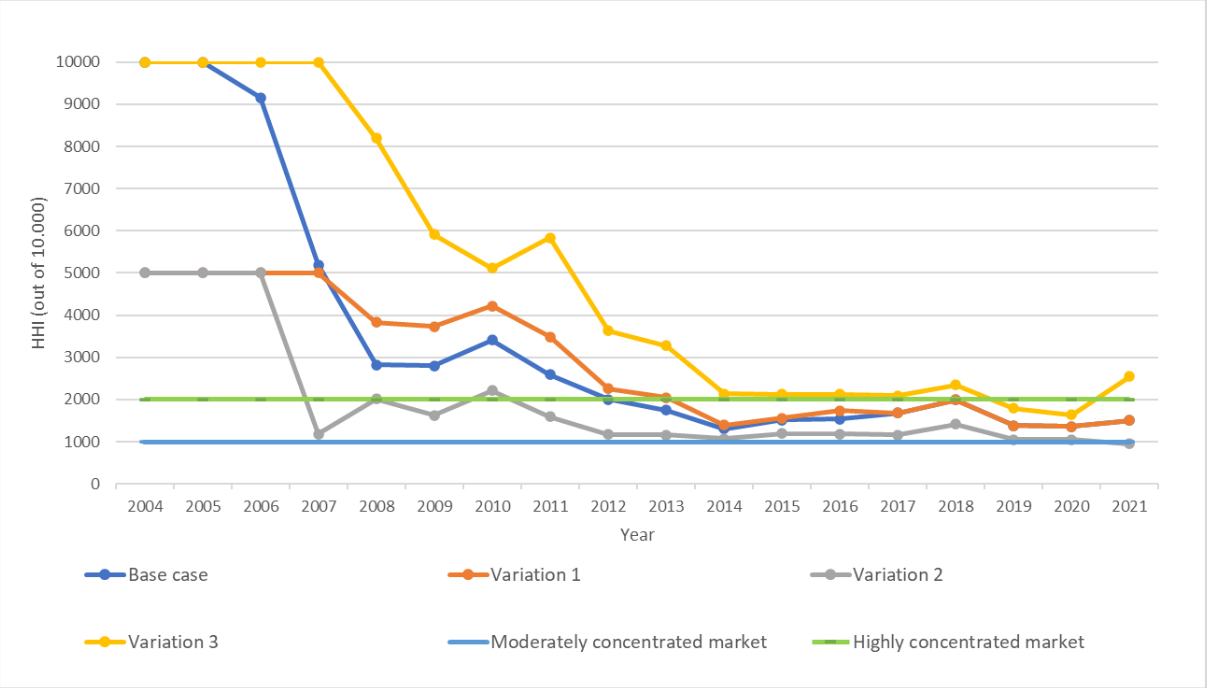

Conversely, where the distribution of nets with unknown supplier takes account of the fact that some suppliers have not been captured in the combined database, market concentration decreases for all years. Even when additional suppliers were included in the sensitivity analysis, however, market concentration did not fall below 1000, which represents a moderately concentrated market. Even when all nets with unknown supplier were allocated to suppliers not included in the combined database, this had very limited impact on the HHI.

Price determinants

Median price was chosen as the response variable as values were not normally distributed and several outliers, including extreme values, were recorded. Optimal model structure and variables to be included were obtained through an iterative process of adding/removing variables, taking into consideration correlation between potential predictors and including interaction terms where relevant. Fixed effects that were explored but did not explain a significant amount of variation in the response variable ($p > 0.05$) were eliminated from the final model. They include ITN procurement channel, lead time and LLIN material (see table S1 above for more detail on database variables).

Figure F2. **Price determinants before and after Global Fund market shaping**. Relationship between real ITN price (USD) and associated price determinants before (panels A and C) and after (panels B and D) implementation of the Global Fund market shaping strategy in 2016.

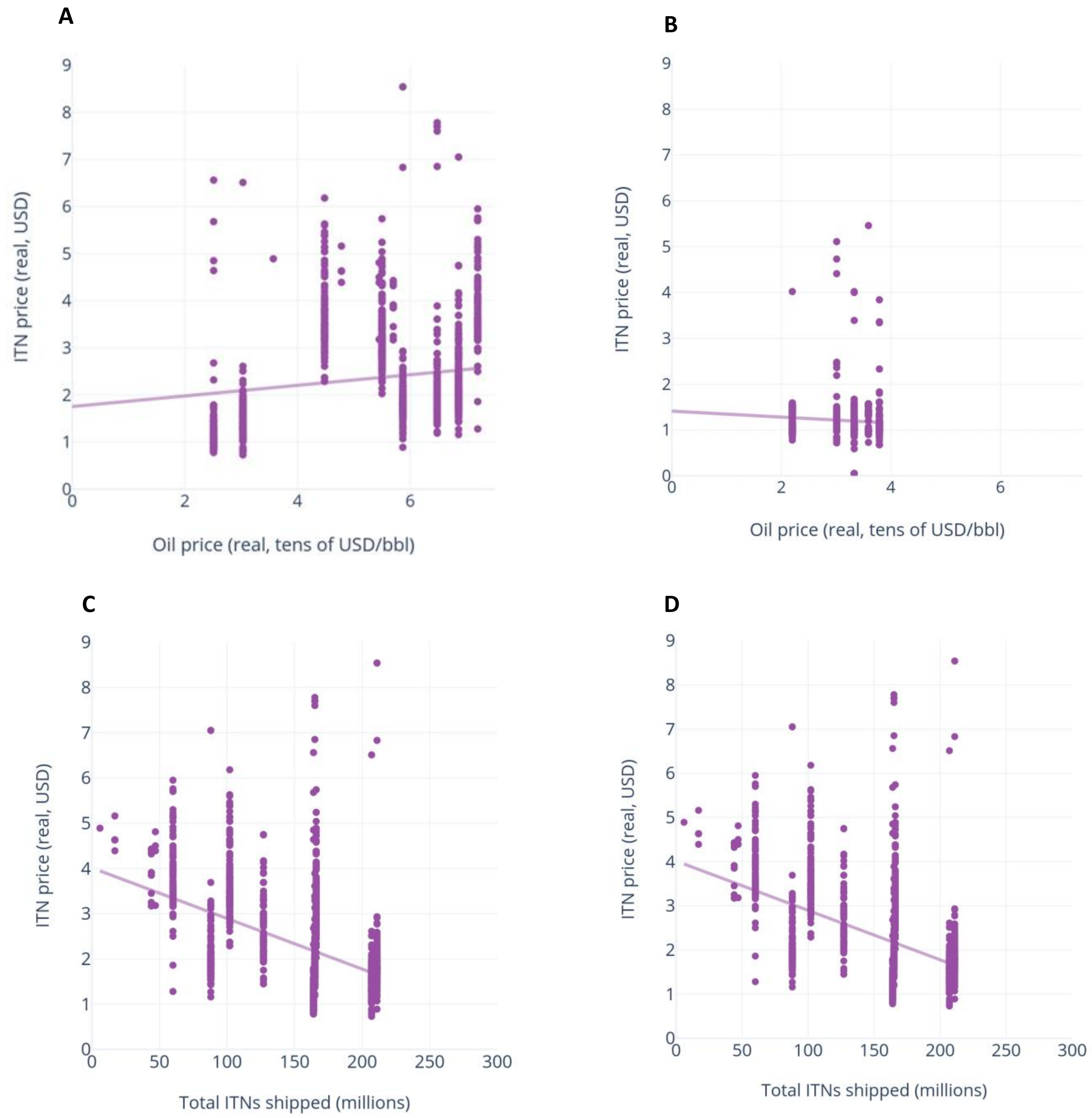

EAC sensitivity analysis

The eighty tornado charts below show for each country and for standard and PBO/dua AI nets the impact on the equivalent annual cost per person protected of varying four main inputs, namely the number of people protected per net, the estimated median retention time, the unit cost of standard and PBO/dual AI nets and the discount rate.

Figure F3 Sensitivity analysis on equivalent annual cost (EAC) per country

A Standard ITNs **B PBO and dual AI ITNs**

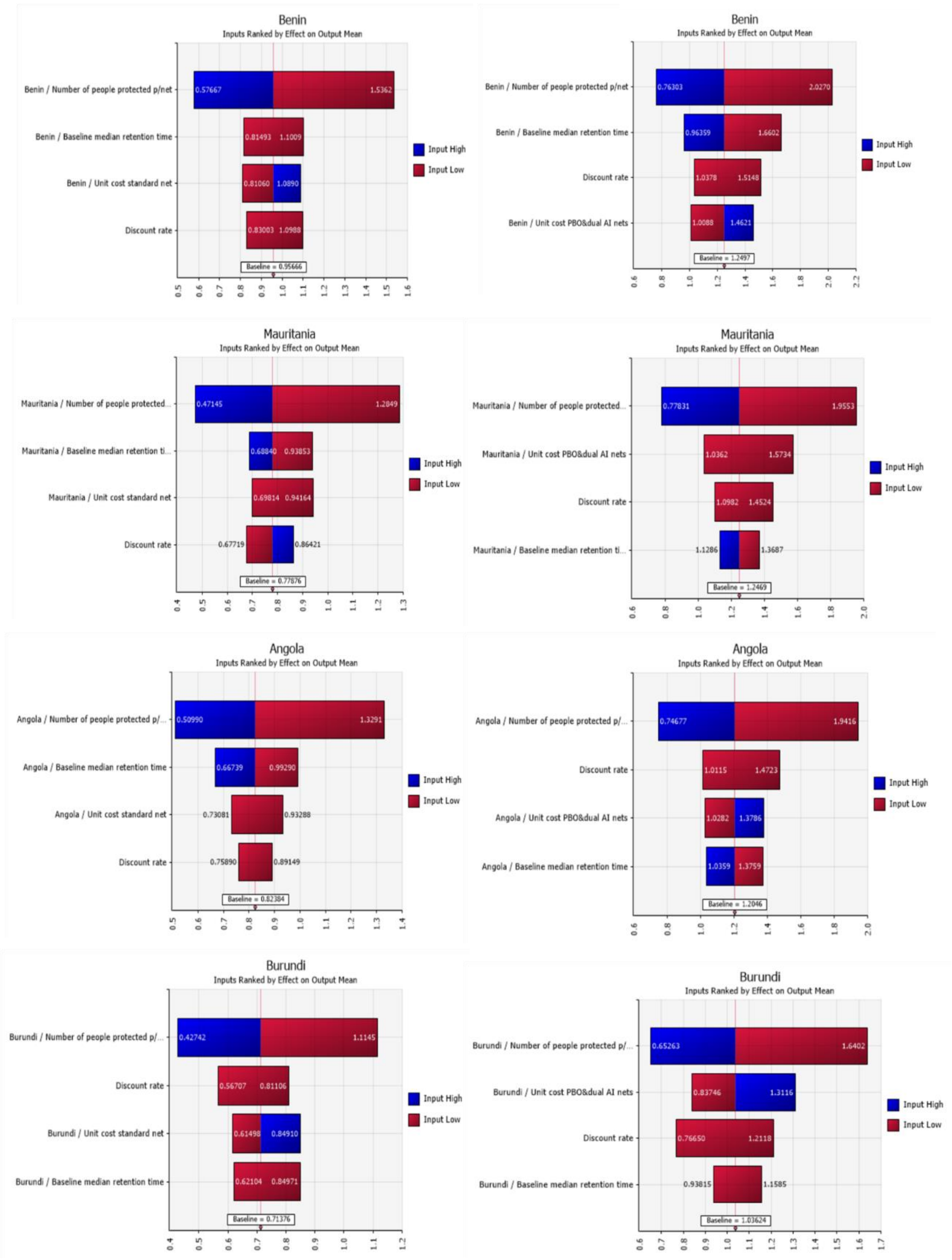

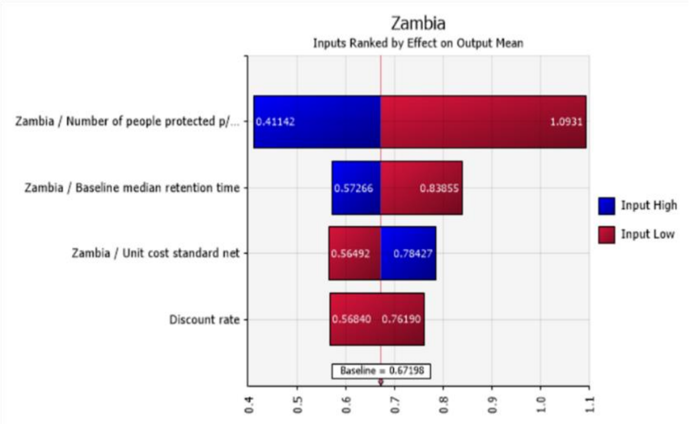
Zambia
Inputs Ranked by Effect on Output Mean
Zambia / Number of people protected p/...
0.41142
1.0931
Zambia / Baseline median retention time
0.57266
0.83855
Zambia / Unit cost standard net
0.56492
0.78427
Discount rate
0.56840
0.76190
Input High
Input Low
Baseline = 0.67198
0.4 0.5 0.6 0.7 0.8 0.9 1.0 1.1

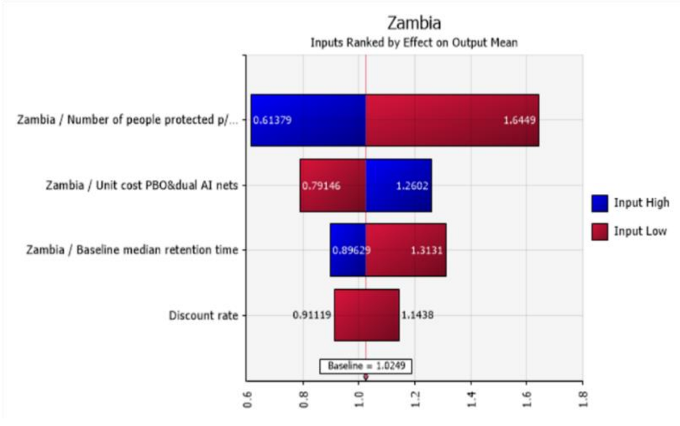
Zambia
Inputs Ranked by Effect on Output Mean
Zambia / Number of people protected p/...
0.61379
1.6449
Zambia / Unit cost PBO&dual AI nets
0.79146
1.2602
Zambia / Baseline median retention time
0.89629
1.3131
Discount rate
0.91119
1.1438
Input High
Input Low
Baseline = 1.0249
0.6 0.8 1.0 1.2 1.4 1.6 1.8

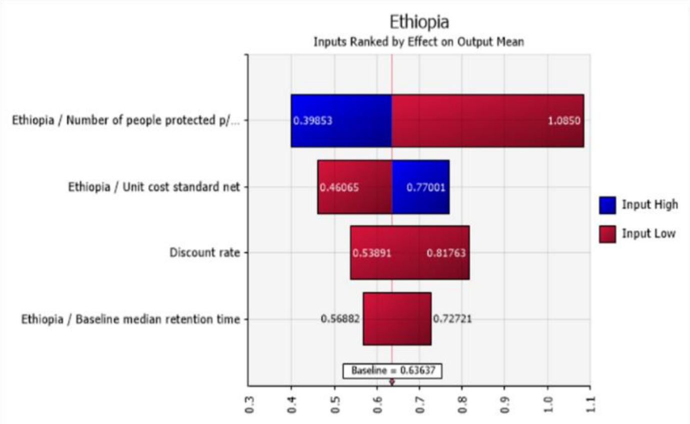
Ethiopia
Inputs Ranked by Effect on Output Mean
Ethiopia / Number of people protected p/...
0.39853
1.0850
Ethiopia / Unit cost standard net
0.46065
0.77001
Discount rate
0.53891
0.81763
Ethiopia / Baseline median retention time
0.56882
0.72721
Input High
Input Low
Baseline = 0.63637
0.3 0.4 0.5 0.6 0.7 0.8 0.9 1.0 1.1

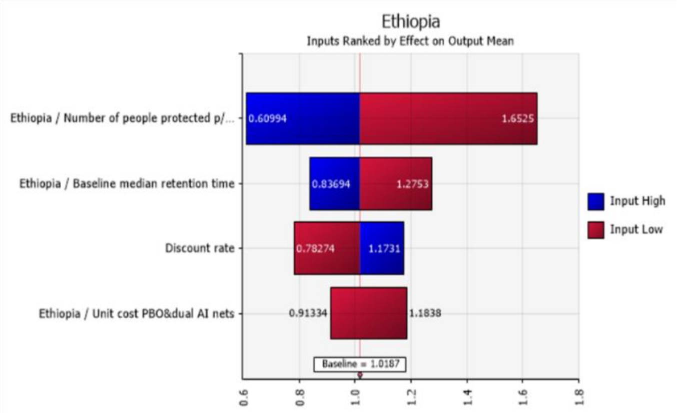
Ethiopia
Inputs Ranked by Effect on Output Mean
Ethiopia / Number of people protected p/...
0.60994
1.6525
Ethiopia / Baseline median retention time
0.83694
1.2753
Discount rate
0.78274
1.1731
Ethiopia / Unit cost PBO&dual AI nets
0.91334
1.1838
Input High
Input Low
Baseline = 1.0187
0.6 0.8 1.0 1.2 1.4 1.6 1.8

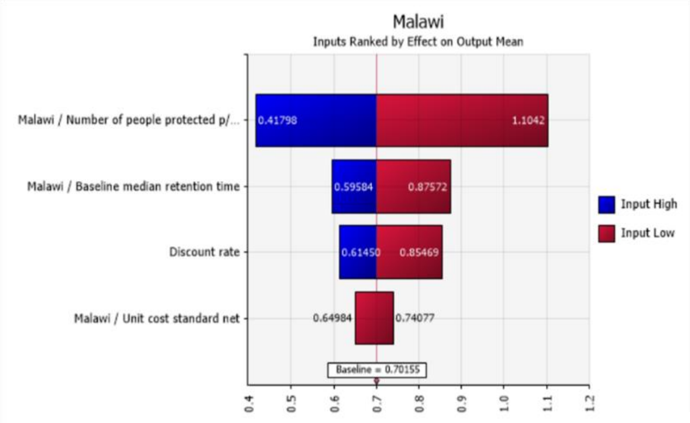
Malawi
Inputs Ranked by Effect on Output Mean
Malawi / Number of people protected p/...
0.41798
1.1042
Malawi / Baseline median retention time
0.59584
0.87572
Discount rate
0.61450
0.85469
Malawi / Unit cost standard net
0.64984
0.74077
Input High
Input Low
Baseline = 0.70155
0.4 0.5 0.6 0.7 0.8 0.9 1.0 1.1 1.2

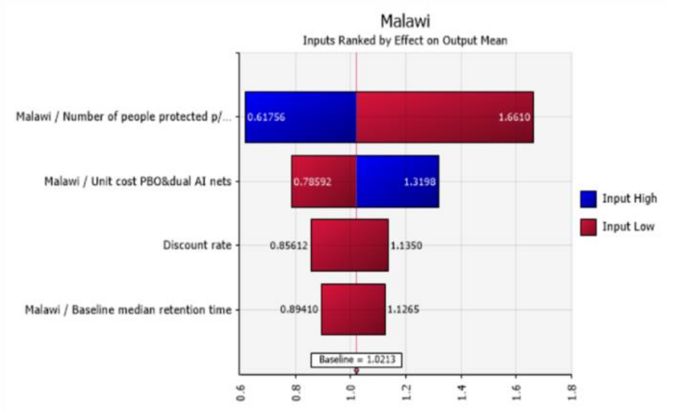
Malawi
Inputs Ranked by Effect on Output Mean
Malawi / Number of people protected p/...
0.61756
1.6610
Malawi / Unit cost PBO&dual AI nets
0.78592
1.3198
Discount rate
0.85612
1.1350
Malawi / Baseline median retention time
0.89410
1.1265
Input High
Input Low
Baseline = 1.0213
0.6 0.8 1.0 1.2 1.4 1.6 1.8

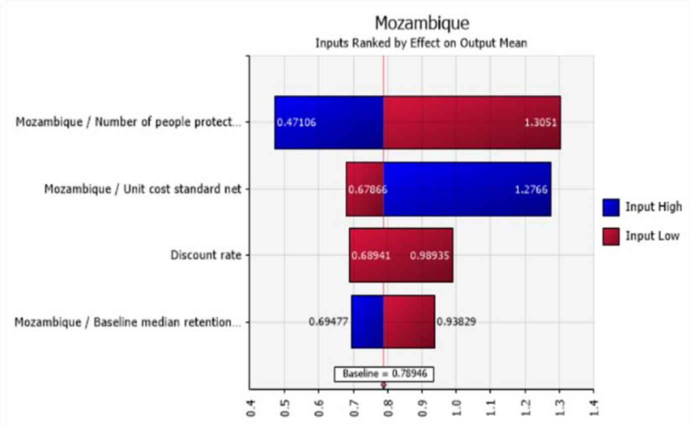
Mozambique
Inputs Ranked by Effect on Output Mean
Mozambique / Number of people protect...
0.47106
1.3051
Mozambique / Unit cost standard net
0.67866
1.2766
Discount rate
0.68941
0.98935
Mozambique / Baseline median retention...
0.69477
0.93829
Input High
Input Low
Baseline = 0.78946
0.4 0.5 0.6 0.7 0.8 0.9 1.0 1.1 1.2 1.3 1.4

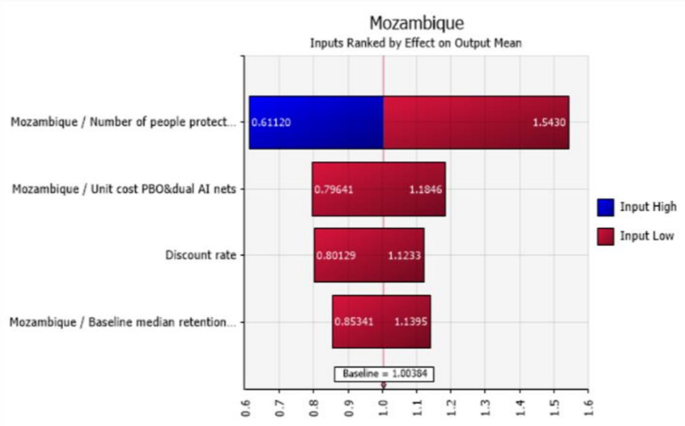
Mozambique
Inputs Ranked by Effect on Output Mean
Mozambique / Number of people protect...
0.61120
1.5430
Mozambique / Unit cost PBO&dual AI nets
0.79641
1.1846
Discount rate
0.80129
1.1233
Mozambique / Baseline median retention...
0.85341
1.1395
Input High
Input Low
Baseline = 1.00384
0.6 0.7 0.8 0.9 1.0 1.1 1.2 1.3 1.4 1.5 1.6

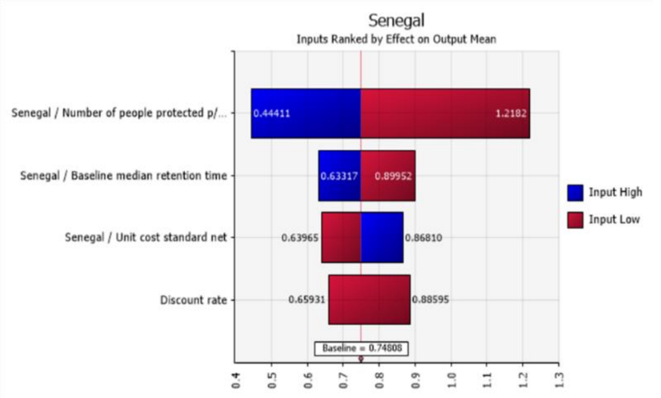
Senegal
Inputs Ranked by Effect on Output Mean
Senegal / Number of people protected p/...
0.44411
1.2182
Senegal / Baseline median retention time
0.63317
0.89952
Senegal / Unit cost standard net
0.63965
0.86810
Discount rate
0.65931
0.88595
Baseline = 0.74808
Input High
Input Low

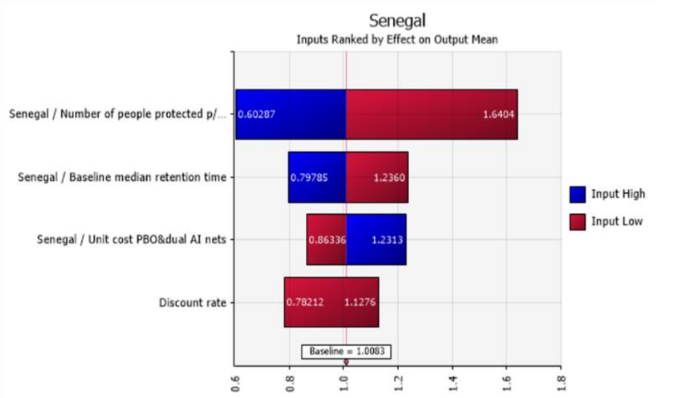
Senegal
Inputs Ranked by Effect on Output Mean
Senegal / Number of people protected p/...
0.60287
1.6404
Senegal / Baseline median retention time
0.79785
1.2360
Senegal / Unit cost PBO&dual AI nets
0.86336
1.2313
Discount rate
0.78212
1.1276
Baseline = 1.0083
Input High
Input Low

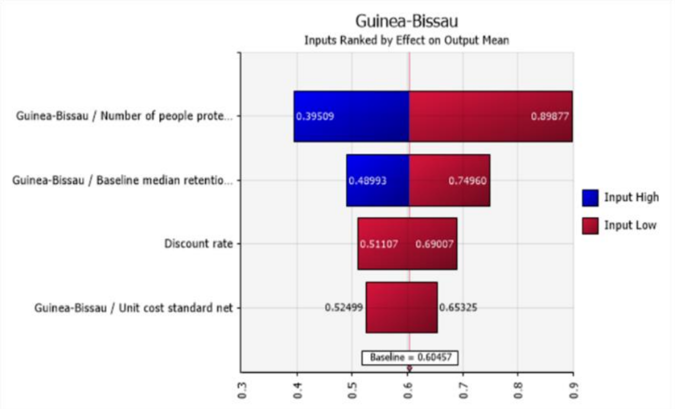
Guinea-Bissau
Inputs Ranked by Effect on Output Mean
Guinea-Bissau / Number of people prote...
0.39509
0.89877
Guinea-Bissau / Baseline median retentio...
0.48993
0.74960
Discount rate
0.51107
0.69007
Guinea-Bissau / Unit cost standard net
0.52499
0.65325
Baseline = 0.60457
Input High
Input Low

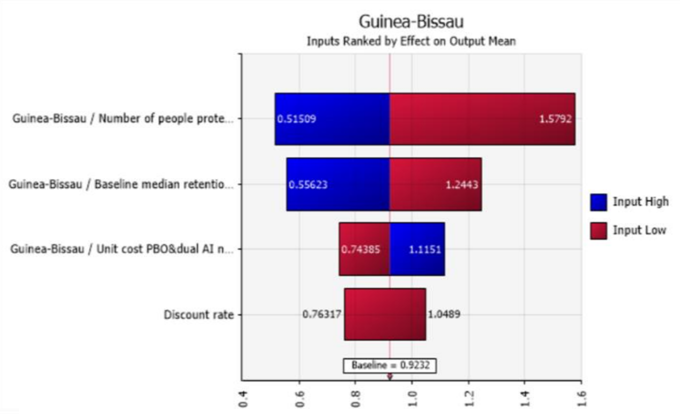
Guinea-Bissau
Inputs Ranked by Effect on Output Mean
Guinea-Bissau / Number of people prote...
0.51509
1.5792
Guinea-Bissau / Baseline median retentio...
0.55623
1.2443
Guinea-Bissau / Unit cost PBO&dual AI n...
0.74385
1.1151
Discount rate
0.76317
1.0489
Baseline = 0.9232
Input High
Input Low

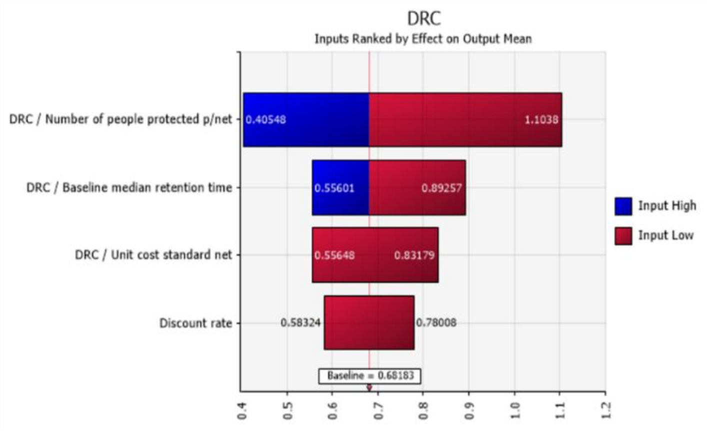
DRC
Inputs Ranked by Effect on Output Mean
DRC / Number of people protected p/net
0.40548
1.1038
DRC / Baseline median retention time
0.55601
0.89257
DRC / Unit cost standard net
0.55648
0.83179
Discount rate
0.58324
0.78008
Baseline = 0.68183
Input High
Input Low

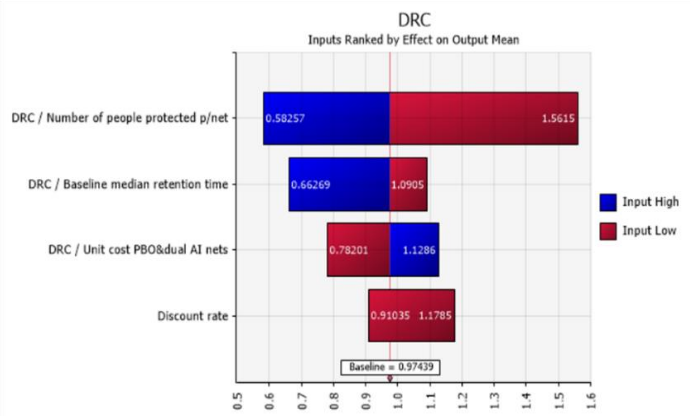
DRC
Inputs Ranked by Effect on Output Mean
DRC / Number of people protected p/net
0.58257
1.5615
DRC / Baseline median retention time
0.66269
1.0905
DRC / Unit cost PBO&dual AI nets
0.78201
1.1286
Discount rate
0.91035
1.1785
Baseline = 0.97439
Input High
Input Low

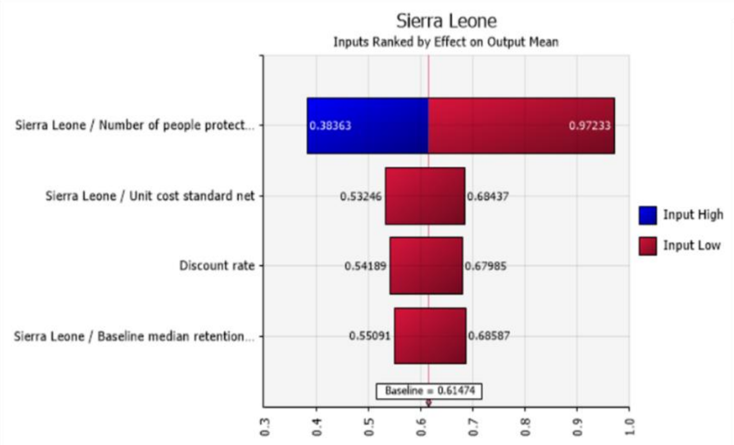
Sierra Leone
Inputs Ranked by Effect on Output Mean
Sierra Leone / Number of people protect...
0.38363
0.97233
Sierra Leone / Unit cost standard net
0.53246
0.68437
Discount rate
0.54189
0.67985
Sierra Leone / Baseline median retention...
0.55091
0.68587
Baseline = 0.61474
Input High
Input Low

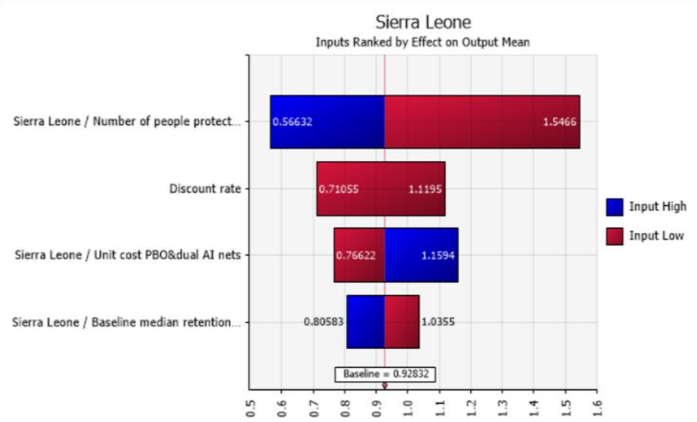
Sierra Leone
Inputs Ranked by Effect on Output Mean
Sierra Leone / Number of people protect...
0.56632
1.5466
Discount rate
0.71055
1.1195
Sierra Leone / Unit cost PBO&dual AI nets
0.76622
1.1594
Sierra Leone / Baseline median retention...
0.80583
1.0355
Baseline = 0.92832
Input High
Input Low

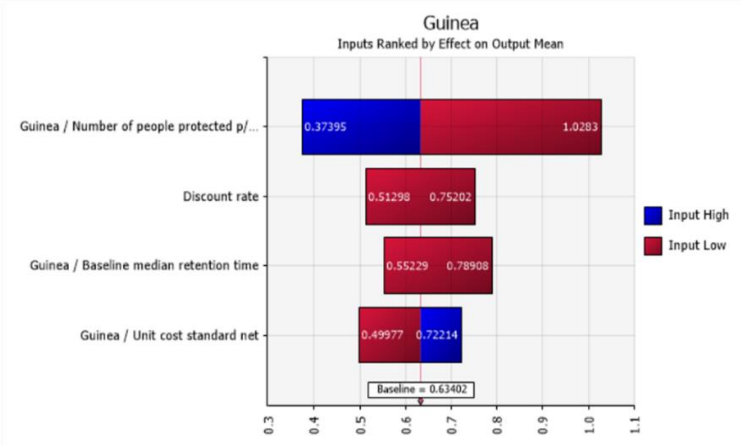
Guinea
Inputs Ranked by Effect on Output Mean
Guinea / Number of people protected p/...
0.37395
1.0283
Discount rate
0.51298
0.75202
Guinea / Baseline median retention time
0.55229
0.78908
Guinea / Unit cost standard net
0.49977
0.72214
Input High
Input Low
Baseline = 0.63402
0.3
0.4
0.5
0.6
0.7
0.8
0.9
1.0
1.1

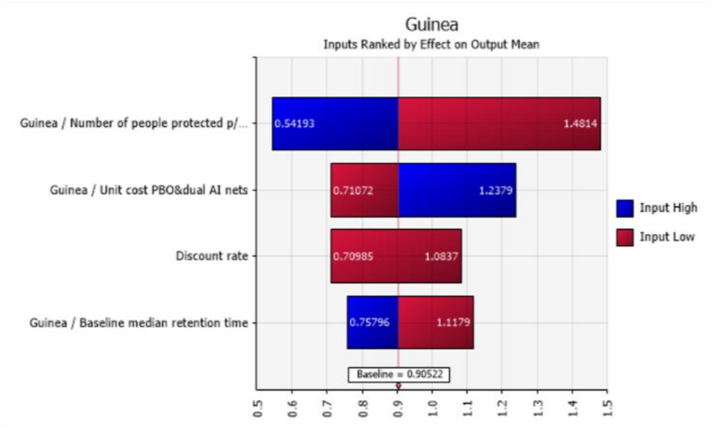
Guinea
Inputs Ranked by Effect on Output Mean
Guinea / Number of people protected p/...
0.54193
1.4814
Guinea / Unit cost PBO&dual AI nets
0.71072
1.2379
Discount rate
0.70985
1.0837
Guinea / Baseline median retention time
0.75796
1.1179
Input High
Input Low
Baseline = 0.90522
0.5
0.6
0.7
0.8
0.9
1.0
1.1
1.2
1.3
1.4
1.5

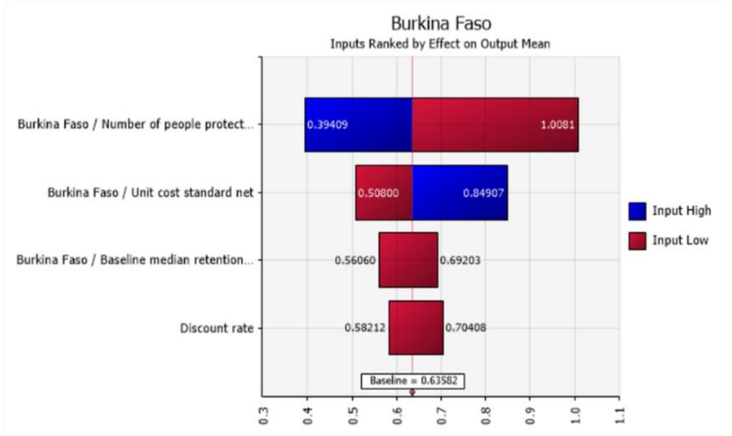
Burkina Faso
Inputs Ranked by Effect on Output Mean
Burkina Faso / Number of people protect...
0.39409
1.0081
Burkina Faso / Unit cost standard net
0.50800
0.84907
Burkina Faso / Baseline median retention...
0.56060
0.69203
Discount rate
0.58212
0.70408
Input High
Input Low
Baseline = 0.63582
0.3
0.4
0.5
0.6
0.7
0.8
0.9
1.0
1.1

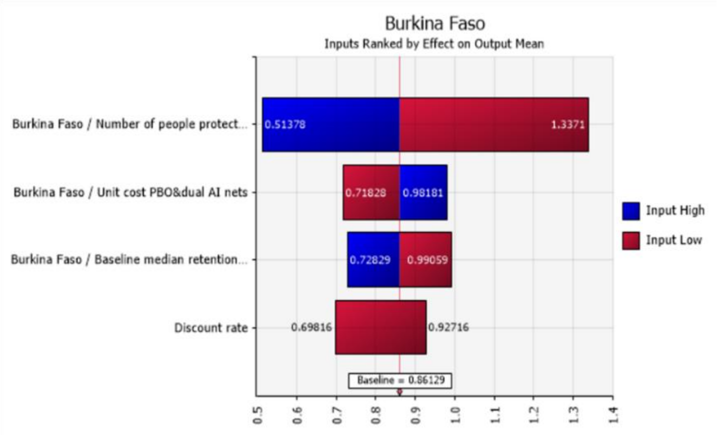
Burkina Faso
Inputs Ranked by Effect on Output Mean
Burkina Faso / Number of people protect...
0.51378
1.3371
Burkina Faso / Unit cost PBO&dual AI nets
0.71828
0.98181
Burkina Faso / Baseline median retention...
0.72829
0.99059
Discount rate
0.69816
0.92716
Input High
Input Low
Baseline = 0.86129
0.5
0.6
0.7
0.8
0.9
1.0
1.1
1.2
1.3
1.4

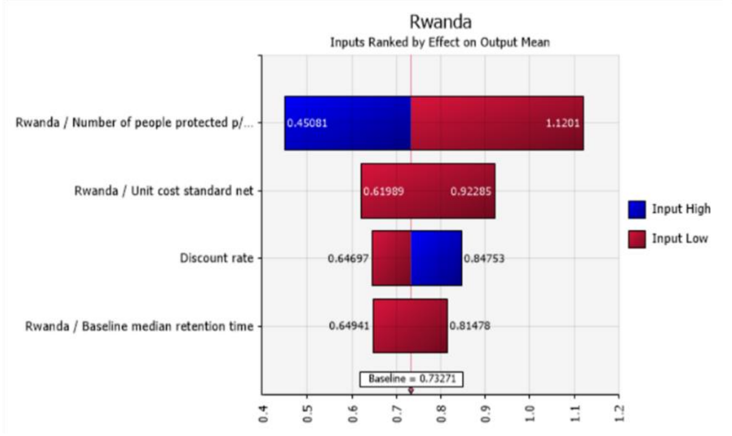
Rwanda
Inputs Ranked by Effect on Output Mean
Rwanda / Number of people protected p/...
0.45081
1.1201
Rwanda / Unit cost standard net
0.61989
0.92285
Discount rate
0.64697
0.84753
Rwanda / Baseline median retention time
0.64941
0.81478
Input High
Input Low
Baseline = 0.73271
0.4
0.5
0.6
0.7
0.8
0.9
1.0
1.1
1.2

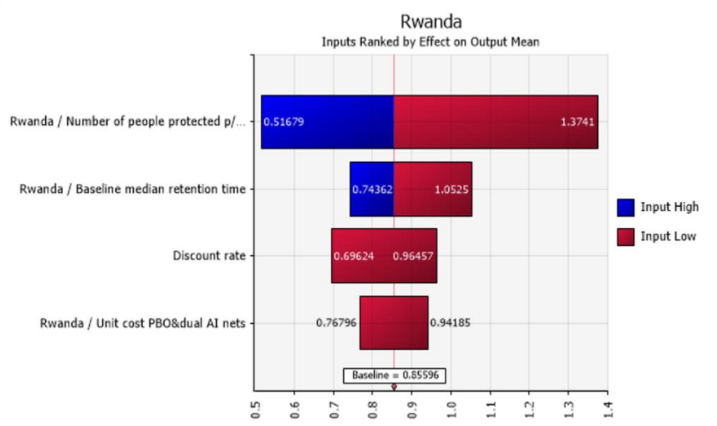
Rwanda
Inputs Ranked by Effect on Output Mean
Rwanda / Number of people protected p/...
0.51679
1.3741
Rwanda / Baseline median retention time
0.74362
1.0525
Discount rate
0.69624
0.96457
Rwanda / Unit cost PBO&dual AI nets
0.76796
0.94185
Input High
Input Low
Baseline = 0.85596
0.5
0.6
0.7
0.8
0.9
1.0
1.1
1.2
1.3
1.4

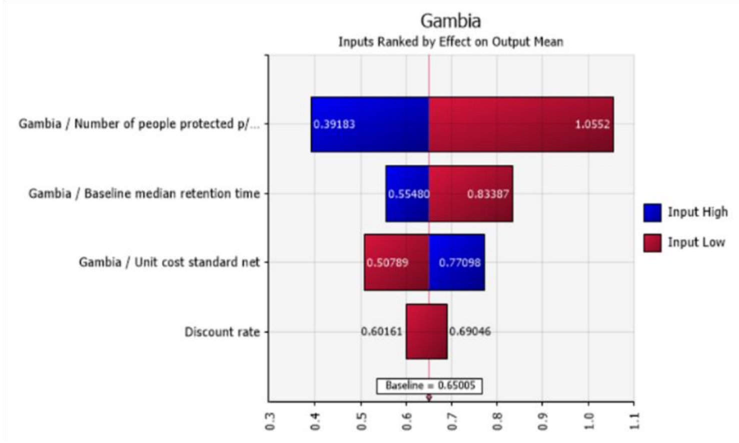
Gambia
Inputs Ranked by Effect on Output Mean
Gambia / Number of people protected p/...
0.39183
1.0552
Gambia / Baseline median retention time
0.55480
0.83387
Gambia / Unit cost standard net
0.50789
0.77098
Discount rate
0.60161
0.69046
Input High
Input Low
Baseline = 0.65005
0.3
0.4
0.5
0.6
0.7
0.8
0.9
1.0
1.1

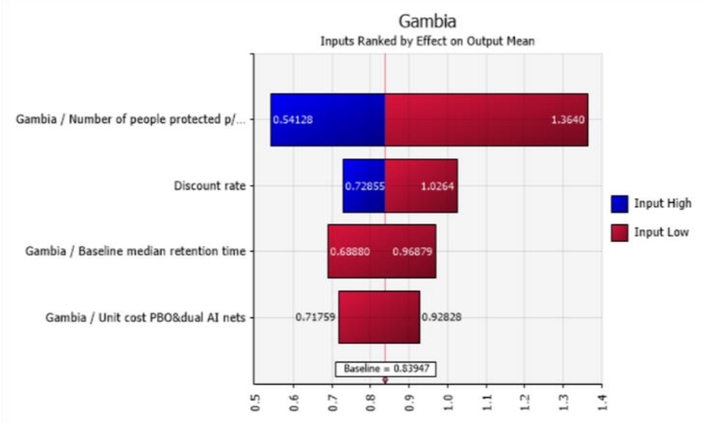
Gambia
Inputs Ranked by Effect on Output Mean
Gambia / Number of people protected p/...
0.54128
1.3640
Discount rate
0.72855
1.0264
Gambia / Baseline median retention time
0.68880
0.96879
Gambia / Unit cost PBO&dual AI nets
0.71759
0.92828
Input High
Input Low
Baseline = 0.83947
0.5
0.6
0.7
0.8
0.9
1.0
1.1
1.2
1.3
1.4

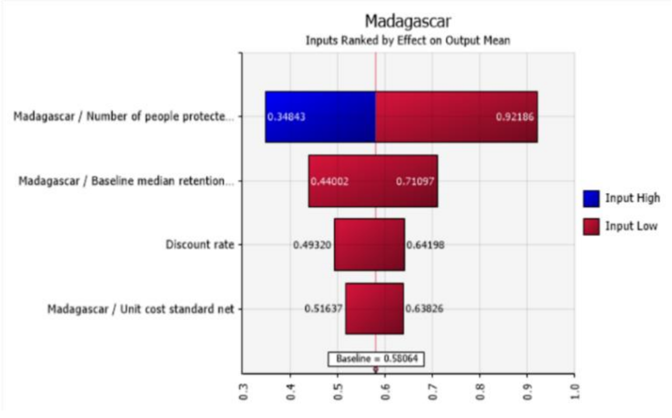
Madagascar
Inputs Ranked by Effect on Output Mean
Madagascar / Number of people protecte...
0.34843
0.92186
Madagascar / Baseline median retention...
0.44002
0.71097
Discount rate
0.49320
0.64198
Madagascar / Unit cost standard net
0.51637
0.63826
Baseline = 0.58064
Input High
Input Low

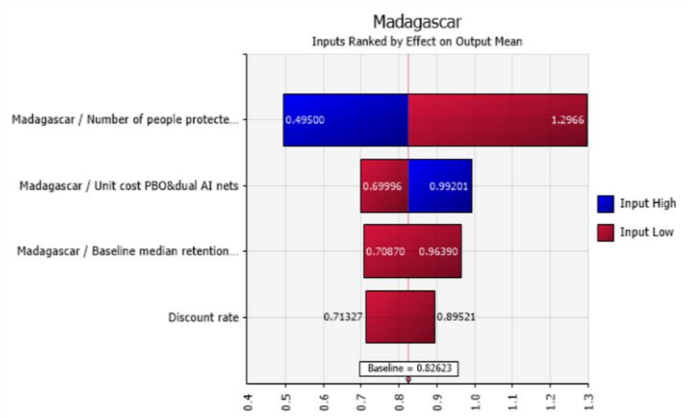
Madagascar
Inputs Ranked by Effect on Output Mean
Madagascar / Number of people protecte...
0.49500
1.2966
Madagascar / Unit cost PBO&dual AI nets
0.69996
0.99201
Madagascar / Baseline median retention...
0.70870
0.96390
Discount rate
0.71327
0.89521
Baseline = 0.82623
Input High
Input Low

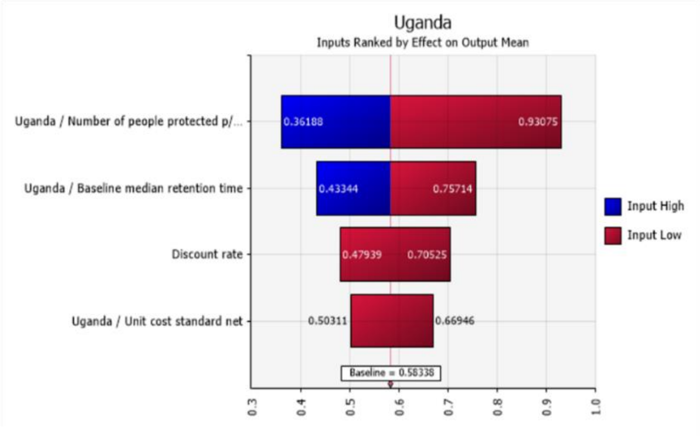
Uganda
Inputs Ranked by Effect on Output Mean
Uganda / Number of people protected p/...
0.36188
0.93075
Uganda / Baseline median retention time
0.43344
0.75714
Discount rate
0.47939
0.70525
Uganda / Unit cost standard net
0.50311
0.66946
Baseline = 0.58338
Input High
Input Low

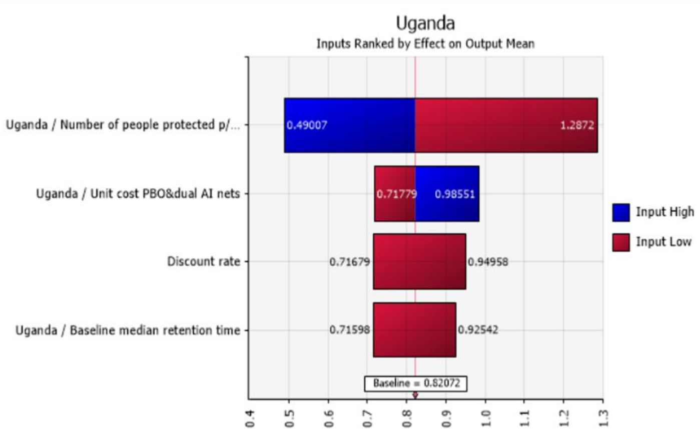
Uganda
Inputs Ranked by Effect on Output Mean
Uganda / Number of people protected p/...
0.49007
1.2872
Uganda / Unit cost PBO&dual AI nets
0.71779
0.98551
Discount rate
0.71679
0.94958
Uganda / Baseline median retention time
0.71598
0.92542
Baseline = 0.82072
Input High
Input Low

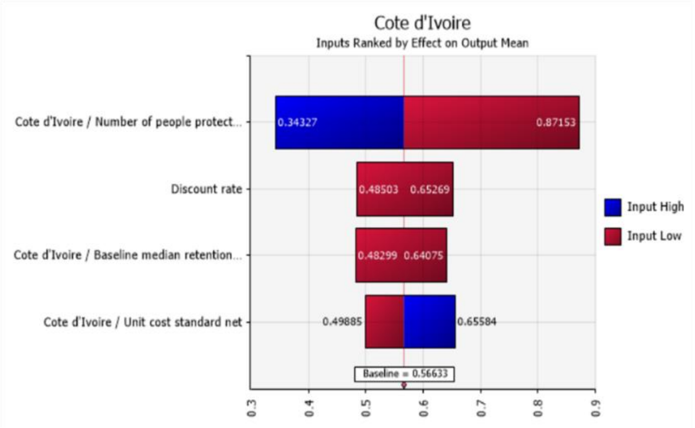
Cote d'Ivoire
Inputs Ranked by Effect on Output Mean
Cote d'Ivoire / Number of people protect...
0.34327
0.87153
Discount rate
0.48503
0.65269
Cote d'Ivoire / Baseline median retention...
0.48299
0.64075
Cote d'Ivoire / Unit cost standard net
0.49885
0.65584
Baseline = 0.56633
Input High
Input Low

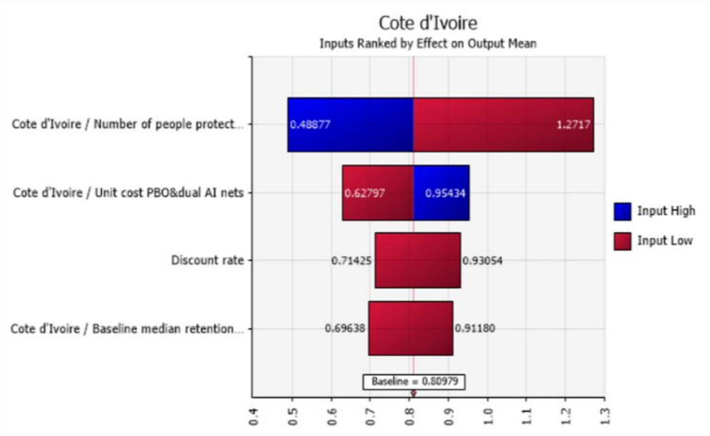
Cote d'Ivoire
Inputs Ranked by Effect on Output Mean
Cote d'Ivoire / Number of people protect...
0.48877
1.2717
Cote d'Ivoire / Unit cost PBO&dual AI nets
0.62797
0.95434
Discount rate
0.71425
0.93054
Cote d'Ivoire / Baseline median retention...
0.69638
0.91180
Baseline = 0.80979
Input High
Input Low

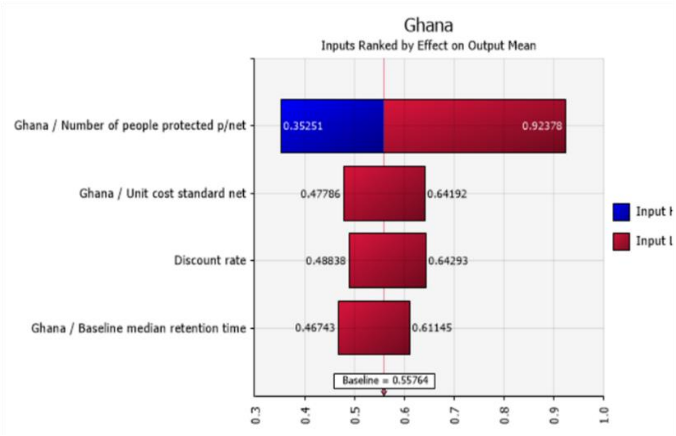
Ghana
Inputs Ranked by Effect on Output Mean
Ghana / Number of people protected p/net
0.35251
0.92378
Ghana / Unit cost standard net
0.47786
0.64192
Discount rate
0.48838
0.64293
Ghana / Baseline median retention time
0.46743
0.61145
Baseline = 0.55764

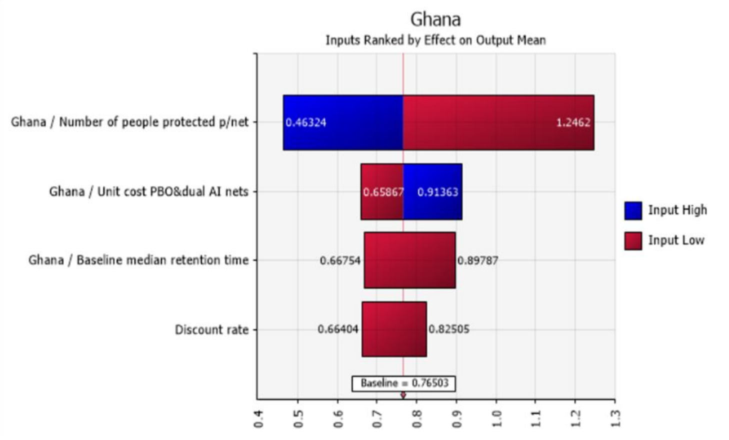
Ghana
Inputs Ranked by Effect on Output Mean
Ghana / Number of people protected p/net
0.46324
1.2462
Ghana / Unit cost PBO&dual AI nets
0.65867
0.91363
Ghana / Baseline median retention time
0.66754
0.89787
Discount rate
0.66404
0.82505
Baseline = 0.76503
Input High
Input Low

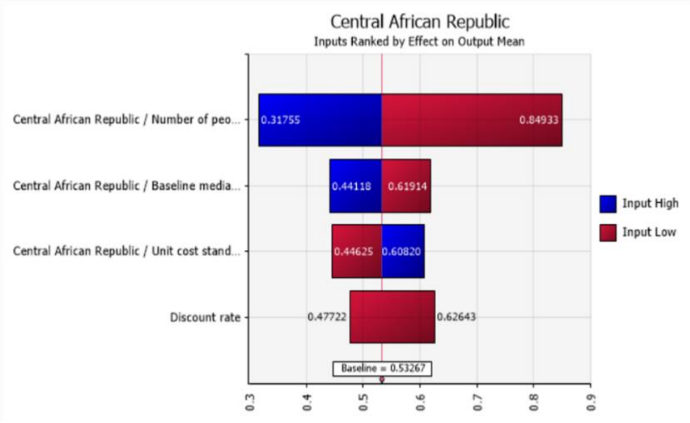
Central African Republic
Inputs Ranked by Effect on Output Mean
Central African Republic / Number of peo...
0.31755
0.84933
Central African Republic / Baseline media...
0.44118
0.61914
Central African Republic / Unit cost stand...
0.44625
0.60820
Discount rate
0.47722
0.62643
Baseline = 0.53267
Input High
Input Low
0.3 0.4 0.5 0.6 0.7 0.8 0.9

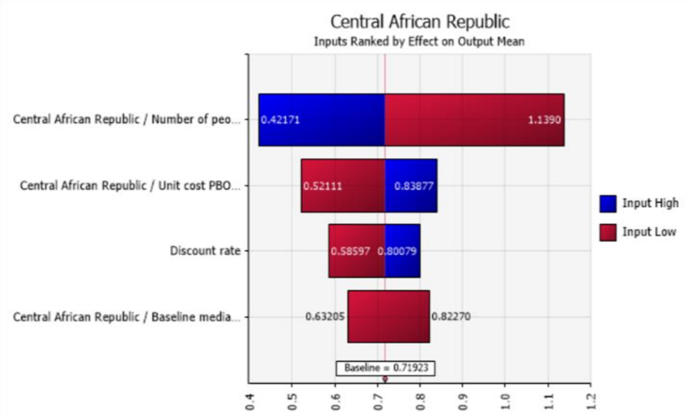
Central African Republic
Inputs Ranked by Effect on Output Mean
Central African Republic / Number of peo...
0.42171
1.1390
Central African Republic / Unit cost PBO...
0.52111
0.83877
Discount rate
0.58597
0.80079
Central African Republic / Baseline media...
0.63205
0.82270
Baseline = 0.71923
Input High
Input Low
0.4 0.5 0.6 0.7 0.8 0.9 1.0 1.1 1.2

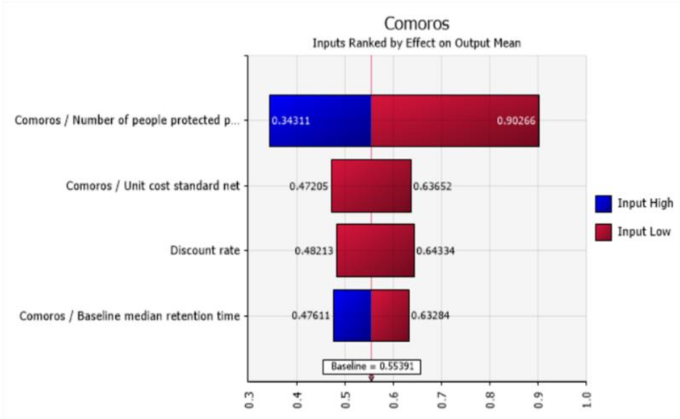
Comoros
Inputs Ranked by Effect on Output Mean
Comoros / Number of people protected p...
0.34311
0.90266
Comoros / Unit cost standard net
0.47205
0.63652
Discount rate
0.48213
0.64334
Comoros / Baseline median retention time
0.47611
0.63284
Baseline = 0.55391
Input High
Input Low
0.3 0.4 0.5 0.6 0.7 0.8 0.9 1.0

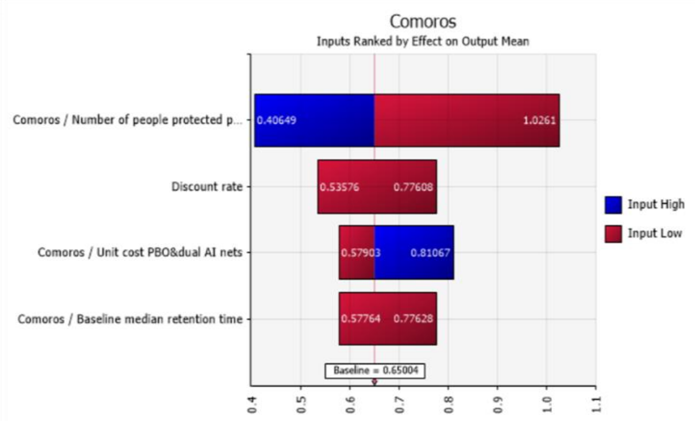
Comoros
Inputs Ranked by Effect on Output Mean
Comoros / Number of people protected p...
0.40649
1.0261
Discount rate
0.53576
0.77608
Comoros / Unit cost PBO&dual AI nets
0.57903
0.81067
Comoros / Baseline median retention time
0.57764
0.77628
Baseline = 0.65004
Input High
Input Low
0.4 0.5 0.6 0.7 0.8 0.9 1.0 1.1

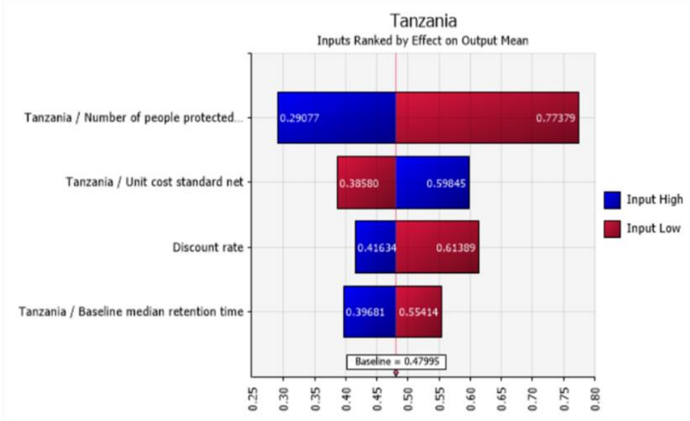
Tanzania
Inputs Ranked by Effect on Output Mean
Tanzania / Number of people protected...
0.29077
0.77379
Tanzania / Unit cost standard net
0.38580
0.59845
Discount rate
0.41634
0.61389
Tanzania / Baseline median retention time
0.39681
0.55414
Baseline = 0.47995
Input High
Input Low
0.25 0.30 0.35 0.40 0.45 0.50 0.55 0.60 0.65 0.70 0.75 0.80

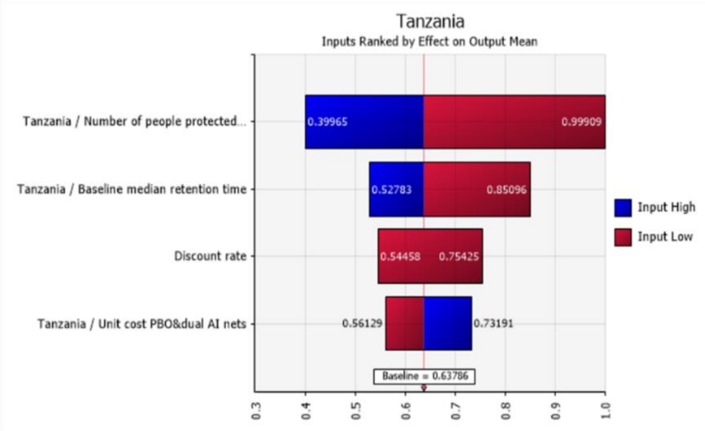
Tanzania
Inputs Ranked by Effect on Output Mean
Tanzania / Number of people protected...
0.39965
0.99909
Tanzania / Baseline median retention time
0.52783
0.85096
Discount rate
0.54458
0.75425
Tanzania / Unit cost PBO&dual AI nets
0.56129
0.73191
Baseline = 0.63786
Input High
Input Low
0.3 0.4 0.5 0.6 0.7 0.8 0.9 1.0

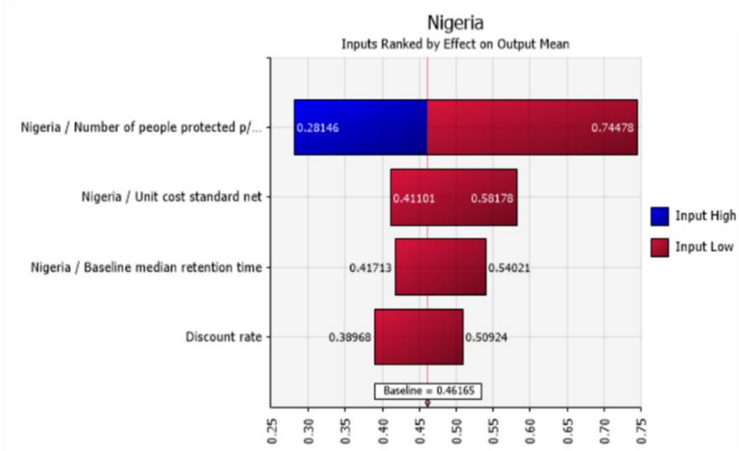
Nigeria
Inputs Ranked by Effect on Output Mean
Nigeria / Number of people protected p/...
0.28146
0.74478
Nigeria / Unit cost standard net
0.41101
0.58178
Nigeria / Baseline median retention time
0.41713
0.54021
Discount rate
0.38968
0.50924
Baseline = 0.46165
Input High
Input Low
0.25 0.30 0.35 0.40 0.45 0.50 0.55 0.60 0.65 0.70 0.75

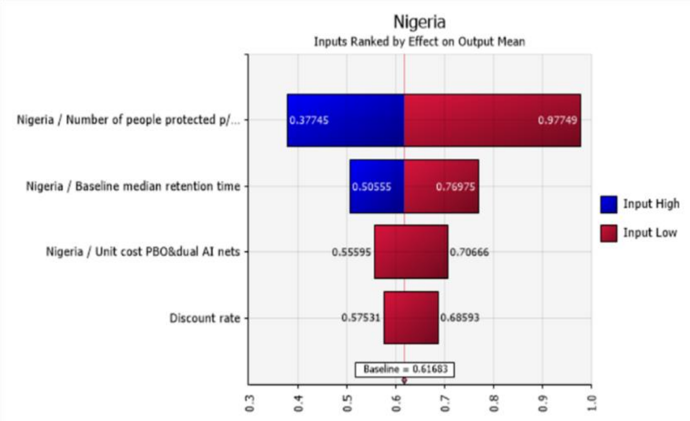
Nigeria
Inputs Ranked by Effect on Output Mean
Nigeria / Number of people protected p/...
0.37745
0.97749
Nigeria / Baseline median retention time
0.50555
0.76975
Nigeria / Unit cost PBO&dual AI nets
0.55595
0.70666
Discount rate
0.57531
0.68593
Baseline = 0.61683
Input High
Input Low
0.3 0.4 0.5 0.6 0.7 0.8 0.9 1.0

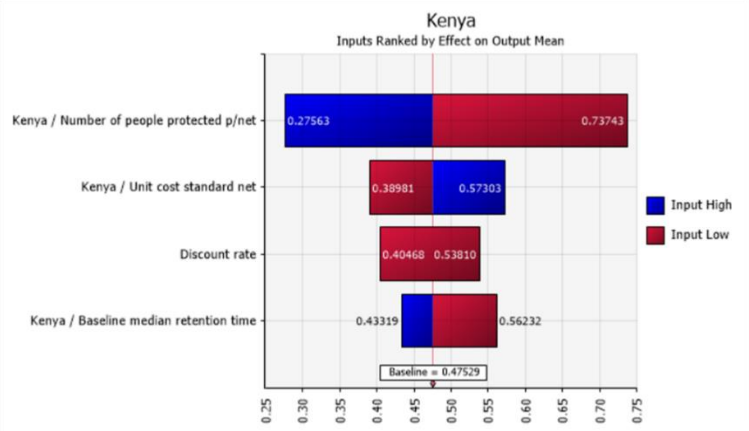
Kenya
Inputs Ranked by Effect on Output Mean
Kenya / Number of people protected p/net
0.27563
0.73743
Kenya / Unit cost standard net
0.38981
0.57303
Discount rate
0.40468
0.53810
Kenya / Baseline median retention time
0.43319
0.56232
Baseline = 0.47529
Input High
Input Low
0.25 0.30 0.35 0.40 0.45 0.50 0.55 0.60 0.65 0.70 0.75

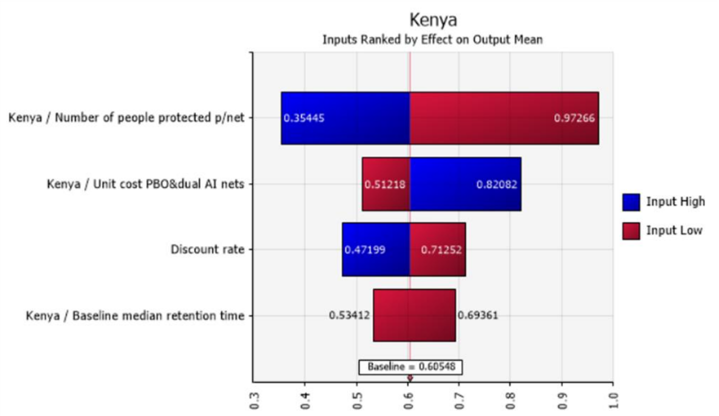
Kenya
Inputs Ranked by Effect on Output Mean
Kenya / Number of people protected p/net
0.35445
0.97266
Kenya / Unit cost PBO&dual AI nets
0.51218
0.82082
Discount rate
0.47199
0.71252
Kenya / Baseline median retention time
0.53412
0.69361
Baseline = 0.60548
Input High
Input Low
0.3 0.4 0.5 0.6 0.7 0.8 0.9 1.0

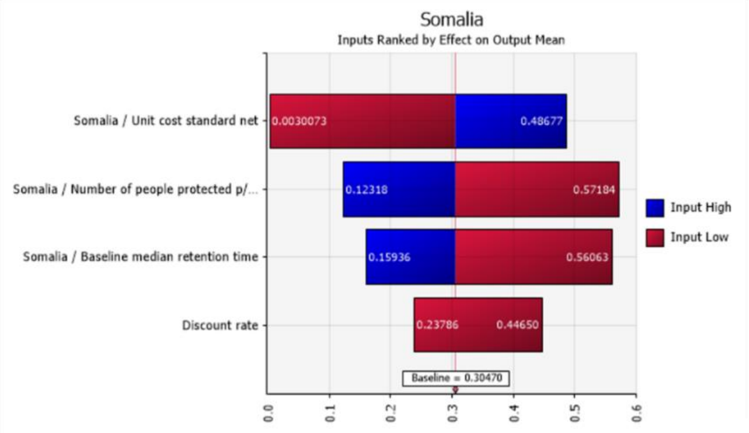
Somalia
Inputs Ranked by Effect on Output Mean
Somalia / Unit cost standard net
0.0030073
0.48677
Somalia / Number of people protected p/...
0.12318
0.57184
Somalia / Baseline median retention time
0.15936
0.56063
Discount rate
0.23786
0.44650
Baseline = 0.30470
Input High
Input Low
0.0 0.1 0.2 0.3 0.4 0.5 0.6

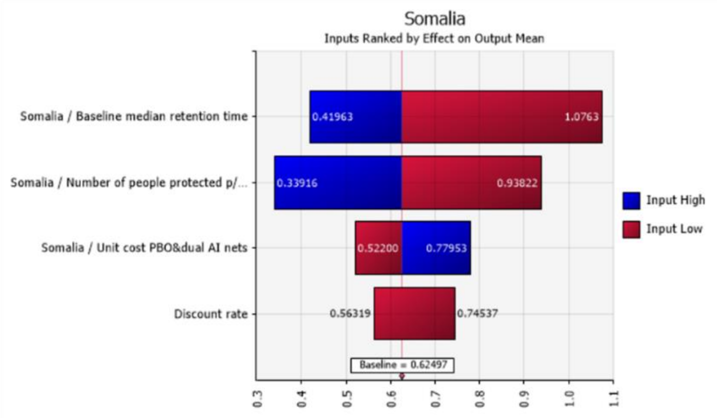
Somalia
Inputs Ranked by Effect on Output Mean
Somalia / Baseline median retention time
0.41963
1.0763
Somalia / Number of people protected p/...
0.33916
0.93822
Somalia / Unit cost PBO&dual AI nets
0.52200
0.77953
Discount rate
0.56319
0.74537
Baseline = 0.62497
Input High
Input Low
0.3 0.4 0.5 0.6 0.7 0.8 0.9 1.0 1.1

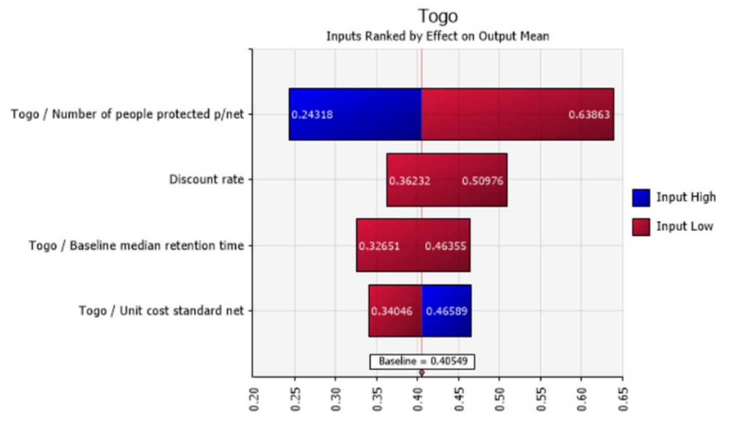
Togo
Inputs Ranked by Effect on Output Mean
Togo / Number of people protected p/net
0.24318
0.63863
Discount rate
0.36232
0.50976
Togo / Baseline median retention time
0.32651
0.46355
Togo / Unit cost standard net
0.34046
0.46589
Baseline = 0.40549
Input High
Input Low
0.20 0.25 0.30 0.35 0.40 0.45 0.50 0.55 0.60 0.65

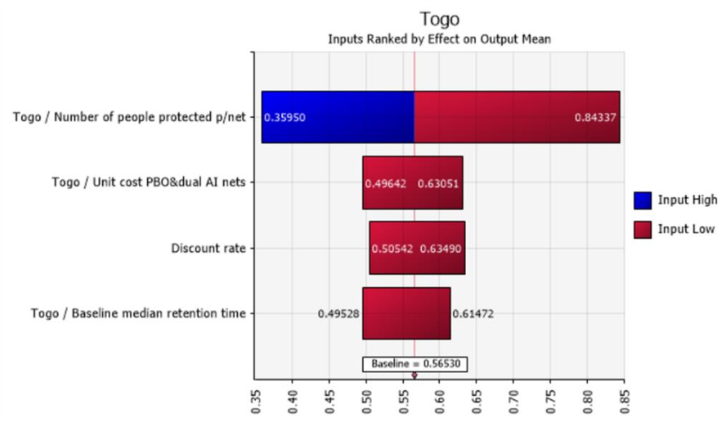
Togo
Inputs Ranked by Effect on Output Mean
Togo / Number of people protected p/net
0.35950
0.84337
Togo / Unit cost PBO&dual AI nets
0.49642
0.63051
Discount rate
0.50542
0.63490
Togo / Baseline median retention time
0.49528
0.61472
Baseline = 0.56530
Input High
Input Low
0.35 0.40 0.45 0.50 0.55 0.60 0.65 0.70 0.75 0.80 0.85

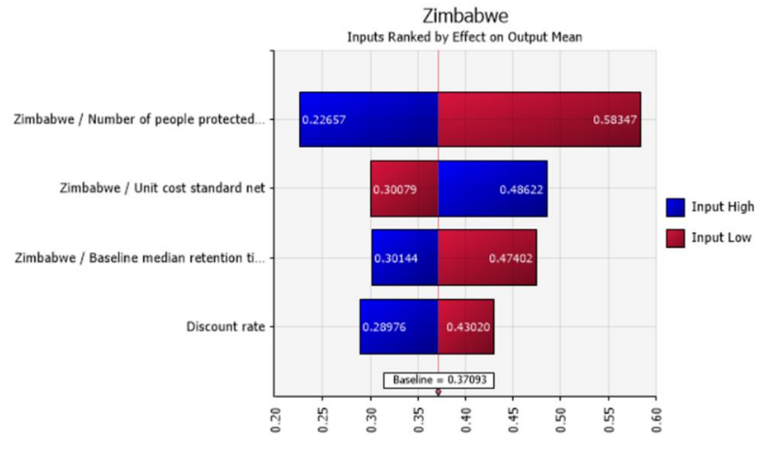
Zimbabwe
Inputs Ranked by Effect on Output Mean
Zimbabwe / Number of people protected...
0.22657
0.58347
Zimbabwe / Unit cost standard net
0.30079
0.48622
Zimbabwe / Baseline median retention ti...
0.30144
0.47402
Discount rate
0.28976
0.43020
Baseline = 0.37093
Input High
Input Low
0.20 0.25 0.30 0.35 0.40 0.45 0.50 0.55 0.60

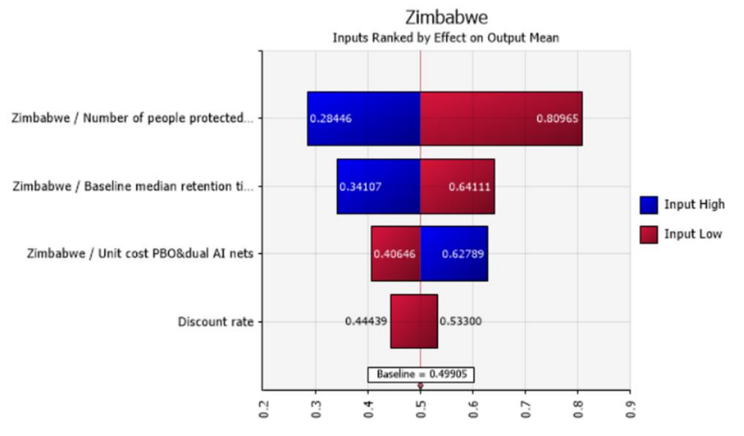
Zimbabwe
Inputs Ranked by Effect on Output Mean
Zimbabwe / Number of people protected...
0.28446
0.80965
Zimbabwe / Baseline median retention ti...
0.34107
0.64111
Zimbabwe / Unit cost PBO&dual AI nets
0.40646
0.62789
Discount rate
0.44439
0.53300
Baseline = 0.49905
Input High
Input Low
0.2 0.3 0.4 0.5 0.6 0.7 0.8 0.9

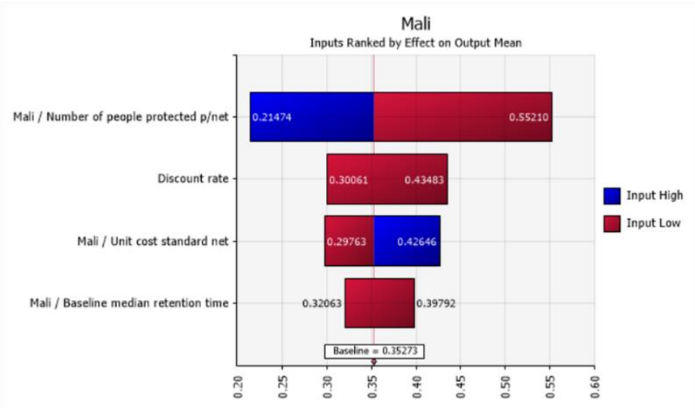
Mali
Inputs Ranked by Effect on Output Mean
Mali / Number of people protected p/net
Discount rate
Mali / Unit cost standard net
Mali / Baseline median retention time
0.21474
0.55210
0.30061
0.43483
0.29763
0.42646
0.32063
0.39792
Baseline = 0.35273
Input High
Input Low
0.20 0.25 0.30 0.35 0.40 0.45 0.50 0.55 0.60

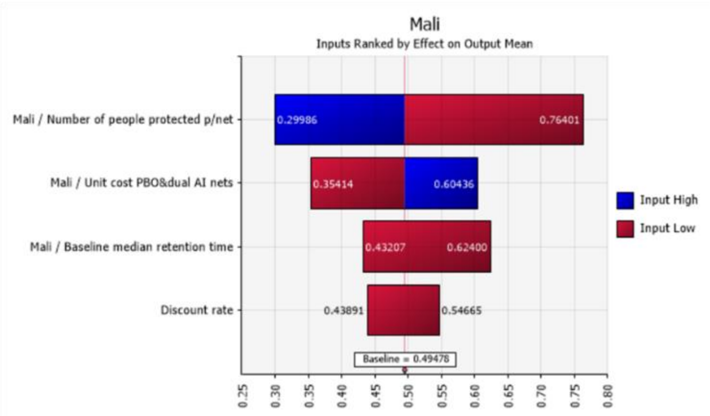
Mali
Inputs Ranked by Effect on Output Mean
Mali / Number of people protected p/net
Mali / Unit cost PBO&dual AI nets
Mali / Baseline median retention time
Discount rate
0.29986
0.76401
0.35414
0.60436
0.43207
0.62400
0.43891
0.54665
Baseline = 0.49478
Input High
Input Low
0.25 0.30 0.35 0.40 0.45 0.50 0.55 0.60 0.65 0.70 0.75 0.80

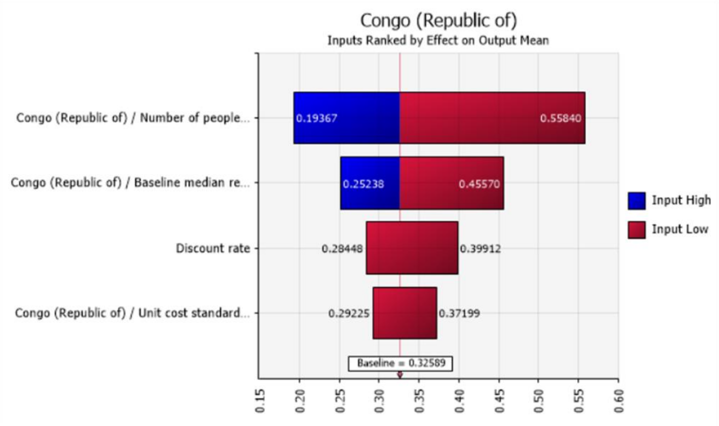
Congo (Republic of)
Inputs Ranked by Effect on Output Mean
Congo (Republic of) / Number of people...
Congo (Republic of) / Baseline median re...
Discount rate
Congo (Republic of) / Unit cost standard...
0.19367
0.55840
0.25238
0.45570
0.28448
0.39912
0.29225
0.37199
Baseline = 0.32589
Input High
Input Low
0.15 0.20 0.25 0.30 0.35 0.40 0.45 0.50 0.55 0.60

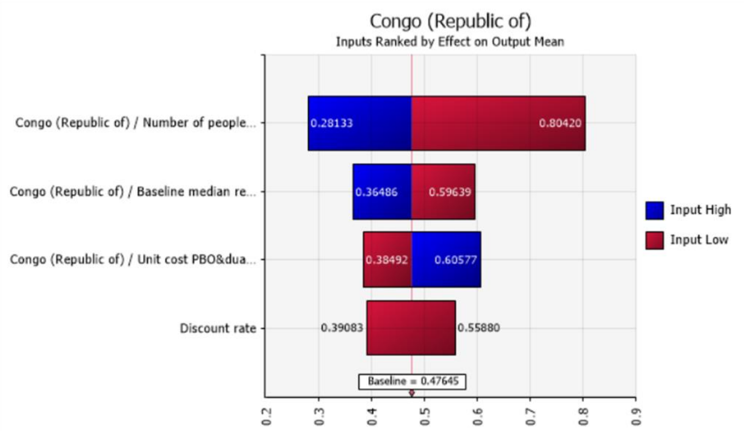
Congo (Republic of)
Inputs Ranked by Effect on Output Mean
Congo (Republic of) / Number of people...
Congo (Republic of) / Baseline median re...
Congo (Republic of) / Unit cost PBO&dua...
Discount rate
0.28133
0.80420
0.36486
0.59639
0.38492
0.60577
0.39083
0.55880
Baseline = 0.47645
Input High
Input Low
0.2 0.3 0.4 0.5 0.6 0.7 0.8 0.9

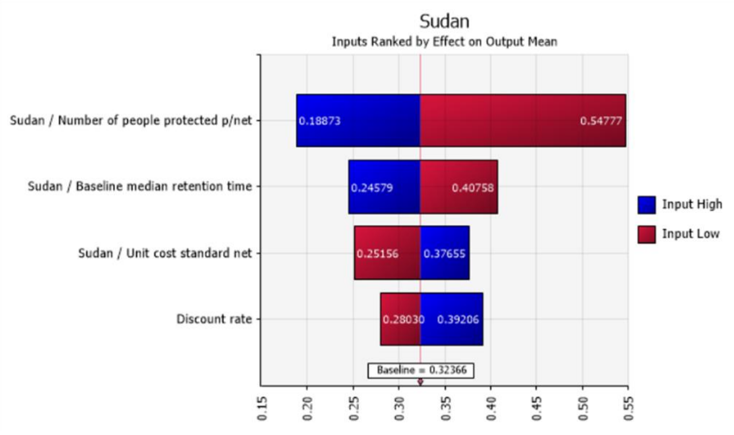
Sudan
Inputs Ranked by Effect on Output Mean
Sudan / Number of people protected p/net
Sudan / Baseline median retention time
Sudan / Unit cost standard net
Discount rate
0.18873
0.54777
0.24579
0.40758
0.25156
0.37655
0.28030
0.39206
Baseline = 0.32366
Input High
Input Low
0.15 0.20 0.25 0.30 0.35 0.40 0.45 0.50 0.55

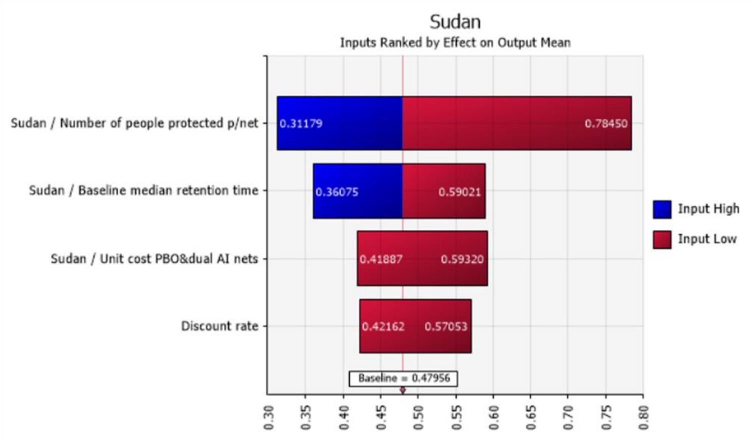
Sudan
Inputs Ranked by Effect on Output Mean
Sudan / Number of people protected p/net
Sudan / Baseline median retention time
Sudan / Unit cost PBO&dual AI nets
Discount rate
0.31179
0.78450
0.36075
0.59021
0.41887
0.59320
0.42162
0.57053
Baseline = 0.47956
Input High
Input Low
0.30 0.35 0.40 0.45 0.50 0.55 0.60 0.65 0.70 0.75 0.80

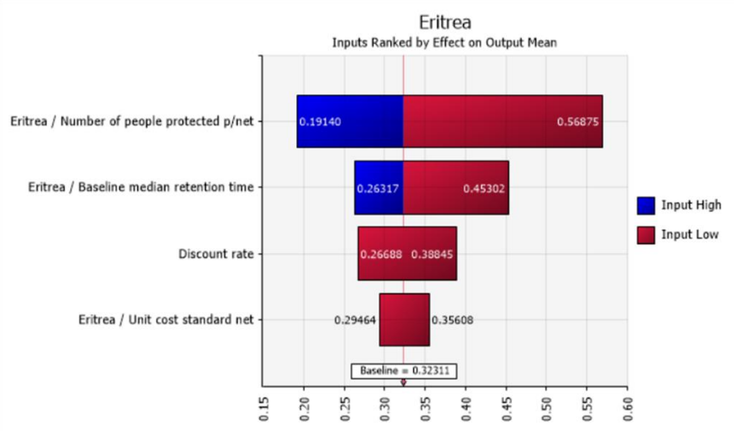
Eritrea
Inputs Ranked by Effect on Output Mean
Eritrea / Number of people protected p/net
Eritrea / Baseline median retention time
Discount rate
Eritrea / Unit cost standard net
0.19140
0.56875
0.26317
0.45302
0.26688
0.38845
0.29464
0.35608
Baseline = 0.32311
Input High
Input Low
0.15 0.20 0.25 0.30 0.35 0.40 0.45 0.50 0.55 0.60

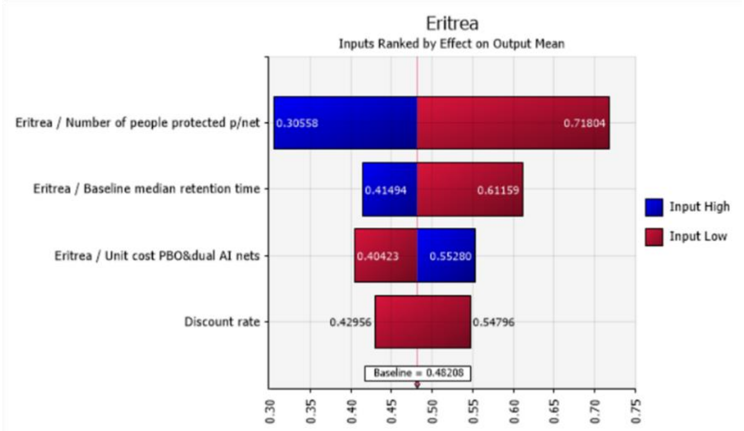
Eritrea
Inputs Ranked by Effect on Output Mean
Eritrea / Number of people protected p/net
Eritrea / Baseline median retention time
Eritrea / Unit cost PBO&dual AI nets
Discount rate
0.30558
0.71804
0.41494
0.61159
0.40423
0.55280
0.42956
0.54796
Baseline = 0.48208
Input High
Input Low
0.30 0.35 0.40 0.45 0.50 0.55 0.60 0.65 0.70 0.75

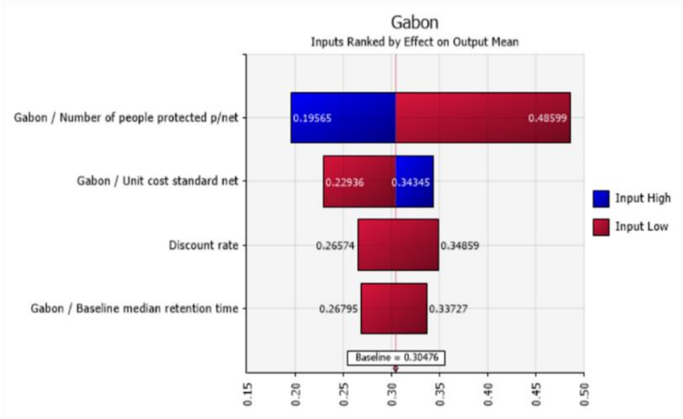
Gabon
Inputs Ranked by Effect on Output Mean
Gabon / Number of people protected p/net
0.19565
0.48599
Gabon / Unit cost standard net
0.22936
0.34345
Discount rate
0.26574
0.34859
Gabon / Baseline median retention time
0.26795
0.33727
Baseline = 0.30476
Input High
Input Low
0.15
0.20
0.25
0.30
0.35
0.40
0.45
0.50

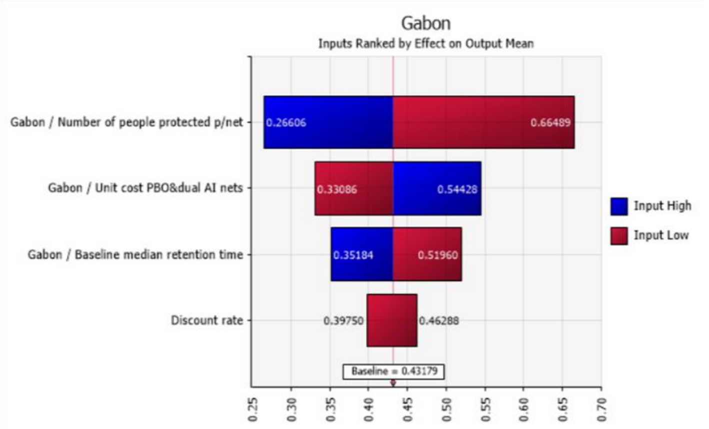
Gabon
Inputs Ranked by Effect on Output Mean
Gabon / Number of people protected p/net
0.26606
0.66489
Gabon / Unit cost PBO&dual AI nets
0.33086
0.54428
Gabon / Baseline median retention time
0.35184
0.51960
Discount rate
0.39750
0.46288
Baseline = 0.43179
Input High
Input Low
0.25
0.30
0.35
0.40
0.45
0.50
0.55
0.60
0.65
0.70

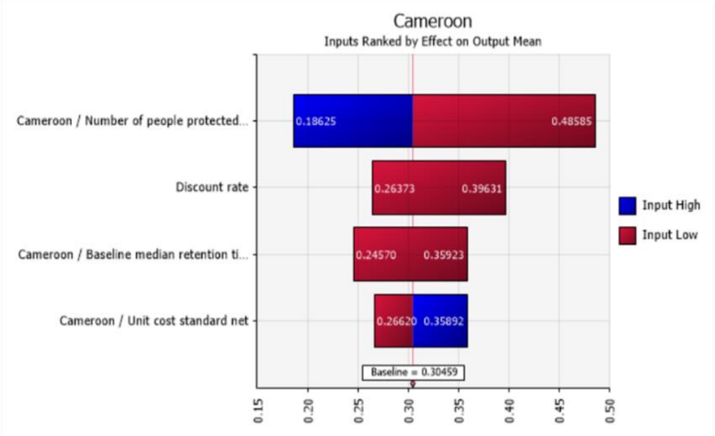
Cameroon
Inputs Ranked by Effect on Output Mean
Cameroon / Number of people protected...
0.18625
0.48585
Discount rate
0.26373
0.39631
Cameroon / Baseline median retention ti...
0.24570
0.35923
Cameroon / Unit cost standard net
0.26620
0.35892
Baseline = 0.30459
Input High
Input Low
0.15
0.20
0.25
0.30
0.35
0.40
0.45
0.50

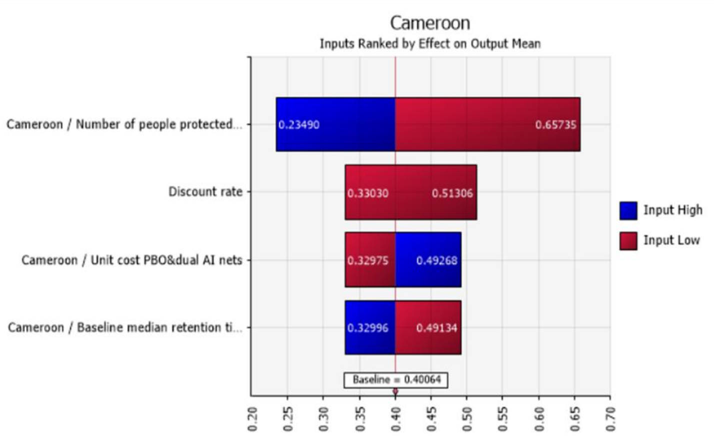
Cameroon
Inputs Ranked by Effect on Output Mean
Cameroon / Number of people protected...
0.23490
0.65735
Discount rate
0.33030
0.51306
Cameroon / Unit cost PBO&dual AI nets
0.32975
0.49268
Cameroon / Baseline median retention ti...
0.32996
0.49134
Baseline = 0.40064
Input High
Input Low
0.20
0.25
0.30
0.35
0.40
0.45
0.50
0.55
0.60
0.65
0.70

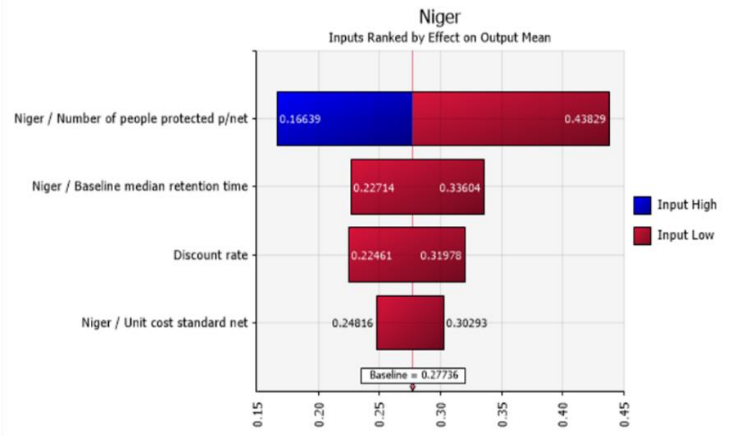
Niger
Inputs Ranked by Effect on Output Mean
Niger / Number of people protected p/net
0.16639
0.43829
Niger / Baseline median retention time
0.22714
0.33604
Discount rate
0.22461
0.31978
Niger / Unit cost standard net
0.24816
0.30293
Baseline = 0.27736
Input High
Input Low
0.15
0.20
0.25
0.30
0.35
0.40
0.45

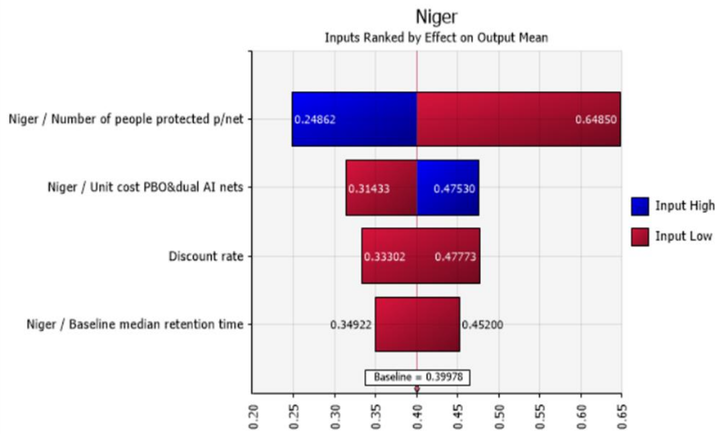
Niger
Inputs Ranked by Effect on Output Mean
Niger / Number of people protected p/net
0.24862
0.64850
Niger / Unit cost PBO&dual AI nets
0.31433
0.47530
Discount rate
0.33302
0.47773
Niger / Baseline median retention time
0.34922
0.45200
Baseline = 0.39978
Input High
Input Low
0.20
0.25
0.30
0.35
0.40
0.45
0.50
0.55
0.60
0.65

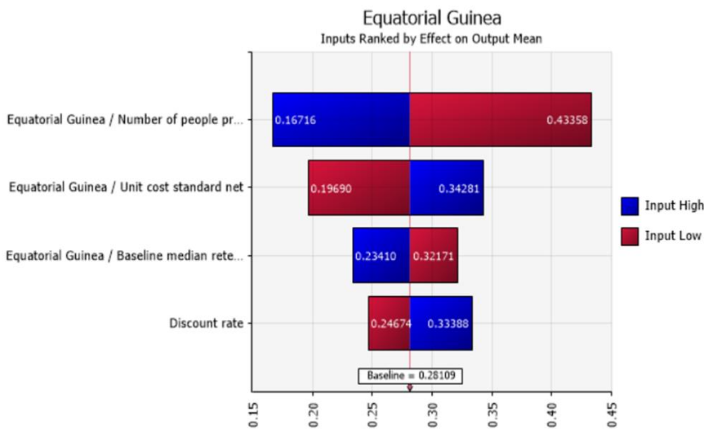
Equatorial Guinea
Inputs Ranked by Effect on Output Mean
Equatorial Guinea / Number of people pr...
0.16716
0.43358
Equatorial Guinea / Unit cost standard net
0.19690
0.34281
Equatorial Guinea / Baseline median rete...
0.23410
0.32171
Discount rate
0.24674
0.33388
Baseline = 0.28109
Input High
Input Low
0.15
0.20
0.25
0.30
0.35
0.40
0.45

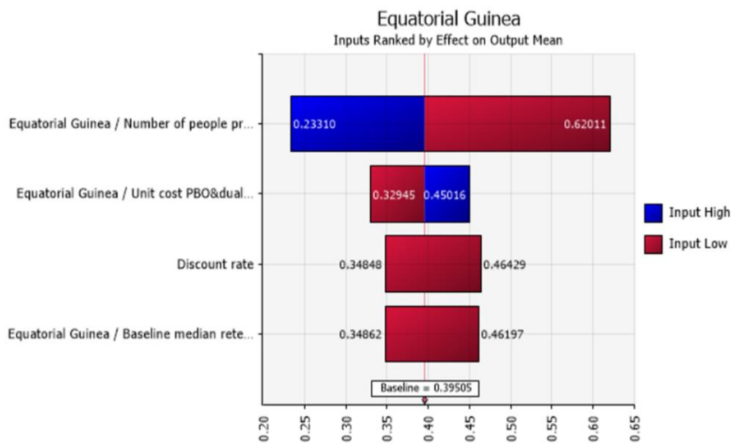
Equatorial Guinea
Inputs Ranked by Effect on Output Mean
Equatorial Guinea / Number of people pr...
0.23310
0.62011
Equatorial Guinea / Unit cost PBO&dual...
0.32945
0.45016
Discount rate
0.34848
0.46429
Equatorial Guinea / Baseline median rete...
0.34862
0.46197
Baseline = 0.39505
Input High
Input Low
0.20
0.25
0.30
0.35
0.40
0.45
0.50
0.55
0.60
0.65

References

1 The Global Fund. Indicative reference costs for budgeting purposes: freight, insurance, and quality assurance. 2021.

2 The World Bank Group. World Development Indicators: Inflation, GDP deflator (annual %). 2021. https://data.worldbank.org/indicator/NY.GDP.DEFL.ZS (accessed Dec 8, 2021).

3 The World Bank Group. World Bank Commodity Price Data (The Pink Sheet). 2022. https://www.worldbank.org/en/research/commodity-markets (accessed Feb 10, 2022).

4 CMA. The State of UK Competition. 2020 www.nationalarchives.gov.uk/doc/open-government-.

5 Bertozzi-Villa A, Bever CA, Koenker H, *et al.* Maps and metrics of insecticide-treated net access, use, and nets-per-capita in Africa from 2000-2020. *Nat Commun* 2021; **12**. DOI:10.1038/s41467-021-23707-7.

6 Kilian A, Koenker H, Paintain L. Estimating population access to insecticide-treated nets from administrative data: Correction factor is needed. *Malar J* 2013; **12**. DOI:10.1186/1475-2875-12-259.

7 Drummond MF, O'Brien B, Stoddart GL, Torrance GW. Methods for the Economic Evaluation of Health Care Programmes, Second Edition. 1997.
